# A C_4_ plant K^+^ channel accelerates stomata to enhance C_3_ photosynthesis and water use efficiency

**DOI:** 10.1093/plphys/kiaf039

**Published:** 2025-01-24

**Authors:** Fernanda A L S Alvim, Jonas Chaves Alvim, Julian M Hibberd, Andrew R Harvey, Michael R Blatt

**Affiliations:** Laboratory of Plant Physiology and Biophysics, Bower Building, University of Glasgow, Glasgow G12 8QQ, UK; Laboratory of Plant Physiology and Biophysics, Bower Building, University of Glasgow, Glasgow G12 8QQ, UK; Department of Plant Sciences, University of Cambridge, Downing Street, Cambridge CB2 3EA, UK; Physics & Astronomy, Kelvin Building, University of Glasgow, Glasgow G12 8QQ, UK; Laboratory of Plant Physiology and Biophysics, Bower Building, University of Glasgow, Glasgow G12 8QQ, UK

## Abstract

Accelerating stomatal kinetics through synthetic optogenetics and mutations that enhance guard cell K^+^ flux has proven a viable strategy to improve water use efficiency and biomass production. Stomata of the model C4 species *Gynandropsis gynandra*, a relative of the C3 plant *Arabidopsis thaliana*, are similarly fast to open and close. We identified and cloned the guard cell rectifying outward K^+^ channel (GROK) of *Gynandropsis* and showed that GROK is preferentially expressed in stomatal guard cells. GROK is homologous to the *Arabidopsis* guard cell K^+^ channel GORK and, expressed in oocytes, yields a K^+^ current consistent with that of Gynandropsis guard cells. Complementing the *Arabidopsis* gork mutant with GROK promoted K^+^ channel gating and K^+^ flux, increasing stomatal kinetics and yielding gains in water use efficiency and biomass with varying light, especially under water limitation. Our findings demonstrate the potential for engineering a C4 K^+^ channel into guard cells of a C3 species, and they speak to the puzzle of how C4 species have evolved mechanisms that enhance water use efficiency and growth under stress.

## Teaser

A K^+^ channel from the C4 plant *Gynandropsis gynandra* accelerates stomatal kinetics, thereby enhancing biomass gains and water use efficiency when expressed in the C3 model *Arabidopsis*.

## Introduction

Stomatal pores form between pairs of guard cells in the leaf epidermis to allow CO_2_ uptake for photosynthetic carbon assimilation. Stomata also provide a pathway for water loss via transpiration, thereby connecting the global carbon and hydrological cycles (Berry et al. 2010; Jasechko et al. 2013; Matthews and Lawson 2019). Guard cells control the stomatal aperture, affecting plant water use, in response to light, changes in CO_2_ within the leaf (pC_i_), atmospheric relative humidity, and water stress (Lawson et al. 2011; Jezek and Blatt 2017; Blatt et al. 2022; Long et al. 2022).

Much effort to improve water use by plants has focused on reducing stomatal densities, despite the penalties in CO_2_ access to the mesophyll for photosynthetic carbon assimilation (Lawson and Blatt 2014; McAusland et al. 2016; Caine et al. 2019). By contrast, accelerating stomatal kinetics in principle circumvents the carbon:water trade-off, especially under fluctuating light typically experienced by plants growing in the field (Lawson and Blatt 2014; Long et al. 2022). As a proof-of-principle, previous work with the synthetic, light-regulated K^+^ channel BLINK1 achieved gains in biomass and reduced water use by speeding stomatal opening to benefit carbon assimilation when light intensities rose, and by accelerating closing to conserve water when light intensities and carbon demand declined (Papanatsiou et al. 2019).

The success of BLINK1 underlines the importance of guard cell ion transport. Indeed, there is a wealth of knowledge of the mechanics and regulation of ion and water transport in these cells. Stomatal aperture is controlled by guard cell turgidity. Guard cells adjust their turgor in response to changes in light and CO_2_ within the leaf, to atmospheric relative humidity, and to the water-stress hormone abscisic acid by transporting solutes, especially K^+^ salts, and water across the plasma membrane (Blatt 2000; Assmann and Jegla 2016; Jezek and Blatt 2017; Zhang et al. 2018). Guard cell H ^+^ -ATPases generate an electrochemical potential difference for H^+^ and a membrane voltage, negative inside, which facilitate K^+^ uptake through K^+^ channels, in *Arabidopsis*, primarily the KAT1 K^+^ channel (Nakamura et al. 1995; Pilot et al. 2001, 2003; Szyroki et al. 2001; Lebaudy et al. 2008) and high-affinity K^+^ transporters (Rodriguez-Navarro et al. 1986; Blatt and Slayman 1987; Blatt and Clint 1989; Maathuis and Sanders 1994; Very et al. 2014). Stomata close when reduced H^+^ pumping and the activation of Cl^−^ channels combine to depolarize the plasma membrane, thereby promoting K^+^ and Cl^−^ efflux from the guard cells.

The GORK K^+^ channel provides a major pathway for K^+^ efflux and eliminating the channel in the *gork* null mutant slows stomatal closure (Ache et al. 2000; Hosy et al. 2003). Like other K^+^ channels mediating K^+^ efflux in plant cells, GORK channel activation—so-called gating—is inhibited by extracellular K^+^ and promoted by depolarizing membrane voltage (Jezek and Blatt 2017). Horaruang et al. (2022) showed that mutations of a putative autoinhibitory domain of GORK reduce inhibition by external K^+^, thereby enhancing channel activity and accelerating stomatal movements. These findings beg the question of whether similar gating characteristics might occur naturally and underpin stomatal responsiveness.

Plants exhibiting C_4_ photosynthesis fix carbon at much lower pC_i_ and require smaller stomatal apertures to support photosynthesis (Sage and Pearcy 2000; Osborne and Sack 2012). Stomata of many C_4_ plants also respond rapidly to changes in light and carbon demand (Osborne and Sack 2012; Ozeki et al. 2022), including those of the model C_4_ plant *Gynandropsis gynandra* (Alvim et al. 2024), a close relative of C_3_ model *Arabidopsis thaliana* (Hoang et al. 2023). Thus, knowledge of the native C_4_ guard cell mechanics might be leveraged to improve the responsiveness of C_3_ plants. Here, we identify the guard cell rectifying outward K^+^ (GROK) channel of *Gynandropsis* to demonstrate that GROK enhances K^+^ flux, and its substitution in *Arabidopsis* in place of the homologous GORK channel is sufficient to promote photosynthetic carbon assimilation and water use efficiency (WUE). Thus, engineering the characteristics of a naturally occurring C_4_ K^+^ channel into the guard cells of a C_3_ species offers gains in WUE and biomass, and it yields insights into the puzzle of how the C_4_ species achieves enhanced stomatal functionality.

## Results

A search of the *Gynandropsis* genome (Hoang et al. 2023) returned 3 candidate genes, Gg13038, Gg10030, and Gg22935, of high homology with the outward-rectifying K^+^ channels GORK and SKOR from the C_3_ relatives *Arabidopsis* and *Tarenaya hassleriana* (Supplementary Table S1 and Supplementary Appendix S1). Of these, Gg13038 was closest phylogenetically to GORK (Supplementary Fig. S1), with 77% amino acid-sequence identity and was predicted to encode a protein of 814 amino acids in length with a molecular mass of 93.8 kDa. Sequence alignment of the protein identified the typical plant cyclic-nucleotide binding-domain (CNBD) K^+^ channel structure (Jegla et al. 2018) including transmembrane segments S1 to S6 with a pore loop between S5 and S6, a C-linker, and a long C-terminal cytosolic sequence comprising cyclic nucleotide binding and ankyrin domains (Supplementary Figs. S2 and S3).

### Heterologous analysis of GROK

**Figure 1.**
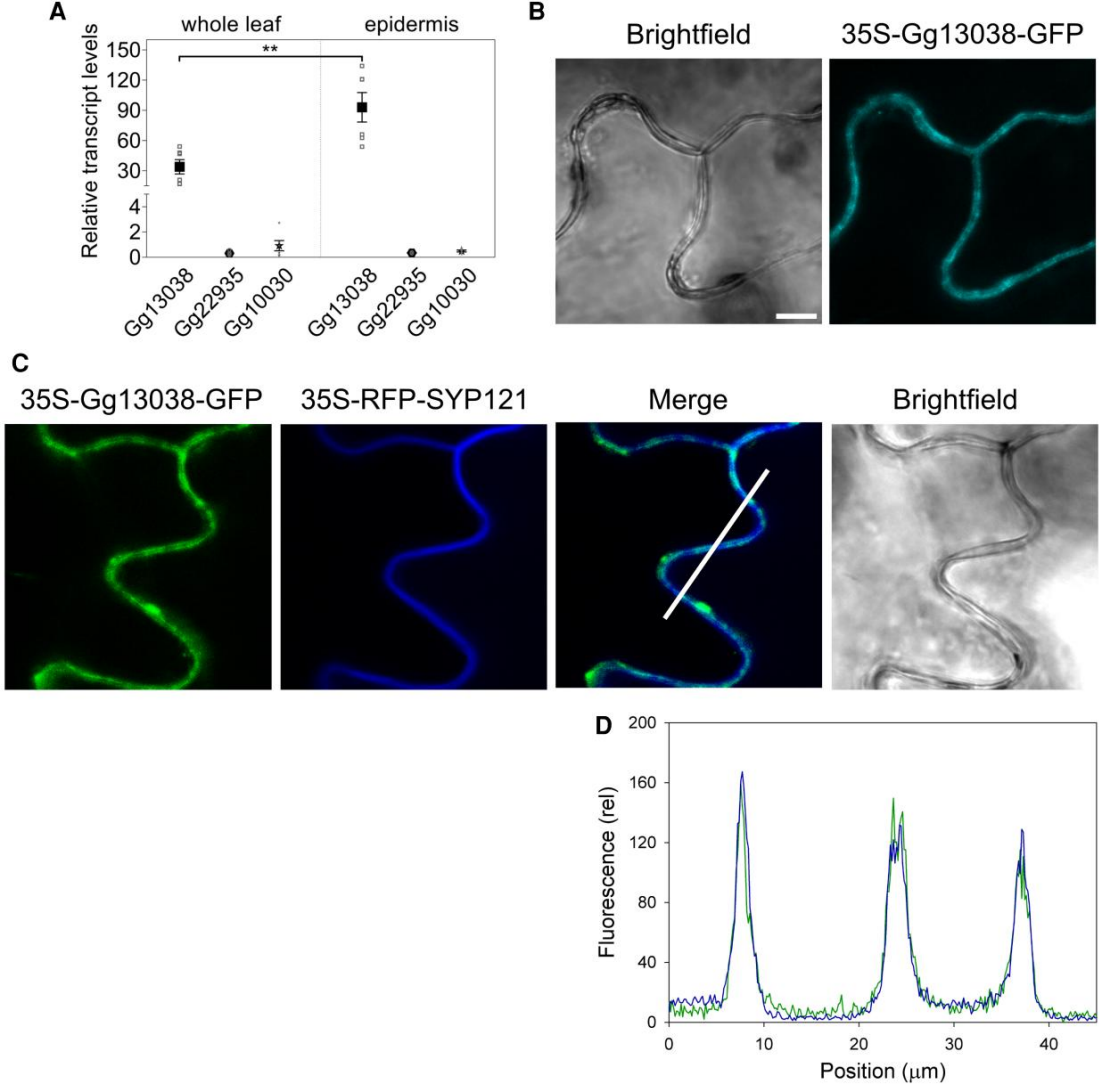
*Gynandropsis gynandra* 13038 localises to the plasma membrane. **A)** Relative transcript levels of *G. gynandra* genes 13038, 22935, and 10030 from whole-leaf and guard cell–enriched epidermal peels. Small symbols are independent experiments; large symbols are means ± SE. Quantitative PCR results normalized to actin 2 gene (Gg00300) with significant differences by post hoc *Tukey* test indicated for Gg13038 (**P < 0.01). **B** and **C)** Confocal laser scanning microscope images of in *Nicotiana tabacum* leaf epidermis after transient transformation to express Gg13038-GFP alone **B)** and with the plasma membrane marker RFP-SYP121 **C)** under control of the constitutive 35S promoter. **C)** Includes the linescan in **D)**. Scale bar **B** and **C)**, 10 µm. **D)** Relative fluorescence intensities for Gg13038-GFP and RFP-SYP121 with position along the linescan in **C)** shows that the 2 fluorescence signals are superimposed.

We isolated *Gynandropsis* leaf epidermis by peeling, which destroys the epidermal cells so that only the guard cells remain intact (Alvim et al. 2024). After quantitative PCR of cDNAs constructed from mRNAs of whole *Gynandropsis* leaves and of the isolated epidermis, we found the Gg13038 gene product to be preferentially expressed in the guard cells (Fig. 1A). *Gynandropsis* does not transform readily. To assess the localization of the putative channel, therefore, we expressed a C-terminal GFP fusion of Gg13038 transiently in tobacco (*Nicotiana tabacum*) epidermis under the control of the constitutive 35S promoter. The protein, hereafter identified as the GROK channel, localized to the plasma membrane together with RFP-tagged SYP121 (Grefen et al. 2010) as a plasma membrane marker (Fig. 1, B to D).

**Figure 2.**
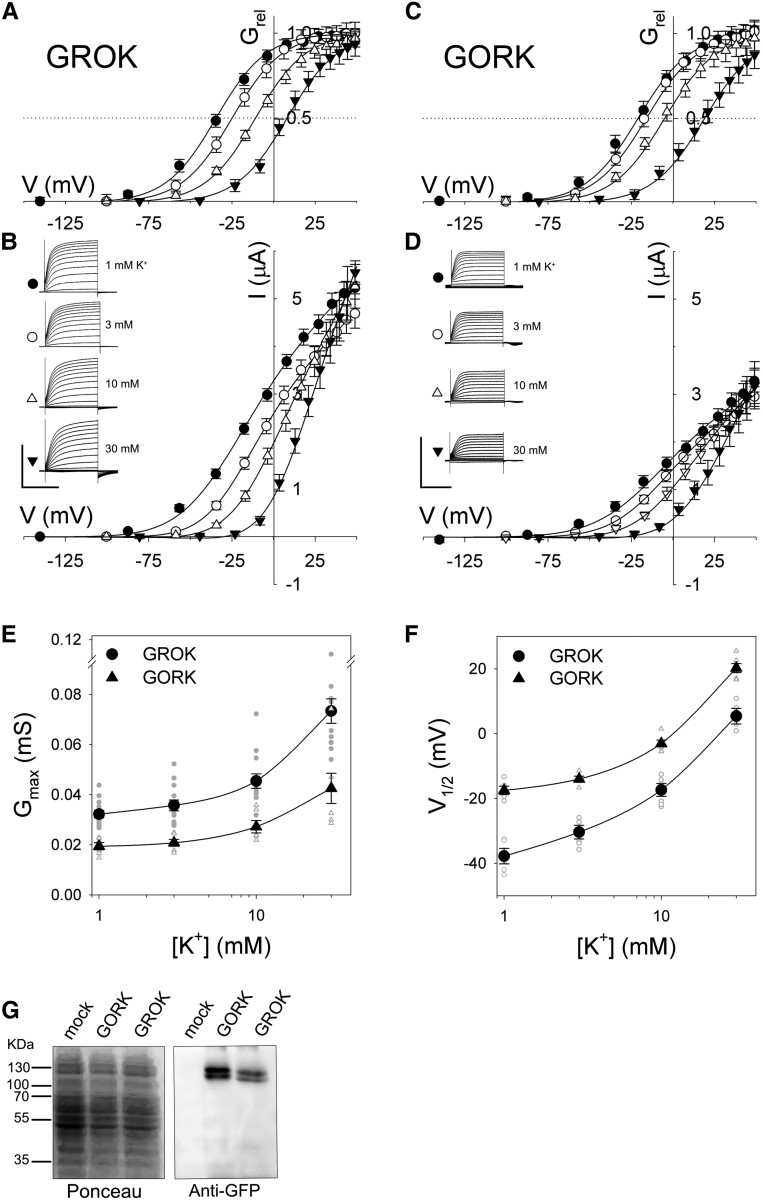
GFP-tagged GROK yields an outward-rectifying K^+^ current. **A** and **B)** Steady-state relative conductance-voltage (G_rel_-V; **A)** and corresponding current-voltage (IV; **B)** curves from *Xenopus* oocytes (*n* = 15 independent experiments) expressing GROK-GFP. Oocytes were superfused with 10 mm HEPES-NaOH, pH 7.3, with 1, 3, 10, and 30 mm KCl, and currents were recorded under voltage clamp. Conditioning voltages, chosen outside the range of channel activity, were −140 mV (1 mm), −100 mV (3 mm), −80 mV (10 mm), and −60 mV (30 mm KCl). Voltage steps (10) ranged from the conditioning voltage to +50 mV and were followed by a voltage step to the conditioning voltage. Insets: representative current traces cross-referenced by symbol. Steady-state IV curves were calculated from currents recorded at the end of the test steps after subtracting the instantaneous background at the beginning of each test step. **C** and **D)** Steady-state G_rel_-V **C)** and corresponding IV **D)** curves from *Xenopus* oocytes (*n* = 6 independent experiments) expressing GORK-GFP. *Insets*: Current traces cross-referenced by symbol. Scale bars **(B** and **D)**: 3 s (horizontal), 4 μA (vertical). Curves **A–D)** are joint, non-linear least-squares fittings to the Boltzmann function (Eqn [1]) and the derived G_rel_ relations (Eqn. [2]) using a Marquardt-Levenberg algorithm; data points **A–D)** are means ± SE of the *n* experiments in each case. Fittings yielded a common δ of 1.7 ± 0.1 for both channels. Dotted lines in **A)** and **C)** indicate the G_rel_ midpoint. Tail current analysis yielded values for K^+^ equilibriium, E_K_, of −56 ± 4 mV for oocytes bathed in 10 mm K^+^ expressing either GORK or GROK. Fittings were therefore carried out assuming [K^+^]_i_ of 100 mm (E_K_ = −60 mV in 10 mm K^+^). Note that varying E_K_ by ±18 mV from −60 mV (2-fold increase and decrease in [K^+^]_i_) had no significant effect on fitted values either for G_max_ or V_1/2_. **E** and **F)** Maximum conductance (G_max_; **E)** and conductance midpoint voltages (V_1/2_; **F)** for GROK-GFP and GORK-GFP. Data from fittings shown **A–D)**. Means ± SE shown as larger symbols and individual data points as smaller symbols. Solid lines are first-order exponential functions and are included as a visual guide only. Significant differences between GORK and GROK were indicated by post hoc *Tukey* test at all K^+^ concentrations for both parameters (*P* < 0.01). **G)** Representative ponceau-stained total membrane SDS gel (left) and immunochemical analysis (right) of oocytes from one set of experiments probed with primary anti-GFP antibodies. The control (mock) was prepared from oocytes injected with water. Molecular weights (kDa, left) and marker for GROK-GFP (center) and GORK-GFP (right) are indicated. Like GORK (Horaruang et al. 2022), GROK appears as a doublet band.

To validate GROK as an outward-rectifying K^+^ channel, we expressed GROK-GFP in *Xenopus* oocytes for functional analysis (Eisenach et al. 2014; Horaruang et al. 2022). Under voltage clamp, GROK-GFP gave a current and conductance (Fig. 2, A and B) qualitatively similar to that in guard cells of *Gynandropsis* (Alvim et al. 2024): stepping from voltages negative of the K^+^ equilibrium voltage (E_K_), to voltages positive of E_K_ yielded an outward current of sigmoidal trajectory and steady-state current-voltage (IV) curves that were displaced to the right with increasing K^+^ outside. A similar current was generated on expressing the *Arabidopsis* GORK channel (Fig. 2, C and D). However, as in vivo (Alvim et al. 2024), the GROK current was typically 50% to 70% greater in amplitude and was evident at more negative voltages when compared with GORK (Fig. 2, A and C,E). The difference in amplitude could not be ascribed to expression of the channel proteins as immunochemical analysis for the GFP tag, a gold-standard for quantifying heterologous expression (Czempinski et al. 2002; Fox et al. 2013; Horaruang et al. 2022), showed a weaker band for GROK expressed in oocytes (Fig. 2G).

We extracted the gating characteristics for the channels by joint fittings of the steady-state current to the Boltzmann function


(1) $$I\;=G_{\max}(V-E_{K})/(1+e^{-\delta F(V-V_{1/2})/\mathrm{RT}}),$$

where G_max_ is the maximum ensemble channel conductance, V is the voltage, E_K_ is the equilibrium voltage for K^+^, V_1/2_ is the voltage yielding the conductance midpoint (=0.5.G_max_), δ is the voltage sensitivity coefficient, and F, R, and T have their usual meanings. We also resolved the gating characteristics of the channels, rearranging Eqn [1] by dividing both sides of the equation by the current component, G_max_(V-E_K_) to give the relative conductance (G_rel_)


(2) $$G_{\mathrm{rel}}=I/(G_{\max}(V-E_{K}))=1/(1+e^{-\delta F(V\;-V_{1/2})/\mathrm{RT}}).$$

As expected, the analysis yielded V_1/2_ values that shifted with K^+^ outside (Fig. 2, A and C,F), as if external K^+^ acted as a voltage-dependent inhibitor of the gate and much as occurs in *Gynandropsis* guard cells (Alvim et al. 2024). It also showed a maximum conductance that increased with K^+^ outside (Fig. 2E). A similar K^+^ dependence was evident for GORK, but the GROK V_1/2_ values were displaced to substantially more negative voltages as they are in vivo, in 1 mm K^+^ by −20 to −25 mV (Fig. 2F). The displacement in V_1/2_ defines the difference in the free energy for gating (ΔΔG) and the affinities (ΔK_aff_) for K^+^ inhibition of the gate


(3) $$\Delta \Delta G=-\delta F(V_{1/2,\mathrm{GORK}}-V_{1/2,\mathrm{GROK}})\;,\mathrm{and}$$

(4) $$\Delta K_{\mathrm{aff}}=\exp(\Delta \Delta G/\mathrm{RT})$$

where V_1/2,GROK_ and V_1/2,GORK_ are the corresponding values for the 2 channels. The free energy change in gating for the GROK was reduced −0.45 Kcal/mol relative to GORK, corresponding to a 1.7-fold lower affinity for K^+^ inhibition. Thus, overall GROK exhibited characteristics that mirrored those in intact *Gynandropsis* guard cells (Alvim et al. 2024).

### Modelling and analysis of GROK in Arabidopsis

Previous work showed that adding a new K^+^ conductance, either using a synthetic, light-activated K^+^ channel (Papanatsiou et al. 2019) or through mutations of a native K^+^ channel (Horaruang et al. 2022), can speed stomata. Horaruang et al. (2022) demonstrated that mutations displacing the V_1/2_ of the *Arabidopsis* GORK channel to more negative voltages accelerated closing and opening, as predicted by OnGuard3 modelling of *Arabidopsis* stomata (Chen et al. 2012; Hills et al. 2012; Jezek et al. 2021). Furthermore, accelerating stomatal kinetics yielded gains in both WUE and biomass under fluctuating light (Horaruang et al. 2022). From the features of GROK in oocytes, it was clear that expression of the channel alone was sufficient to account for similar, negative-shifted values of V_1/2_. We therefore asked whether displacing V_1/2_ by −25 mV relative to GORK might be sufficient to speed stomatal movements, with or without an increase in G_max_.

Incorporating parameters for GROK gating in OnGuard3 simulations of *Arabidopsis* (Supplementary Figs. S4 and S5) predicted a small inward current through the channel near and negative of E_K_; it indicated a significant increase in the speed of stomatal closing both with a step down in light and with a step up in the partial pressure of CO_2_; and the simulations predicted a small increase in the speed of reopening when either the light or CO_2_ step was reversed. In each case, the accelerations in stomatal kinetics were a direct consequence of the additional K^+^ flux arising with the shift in V_1/2_. Trials with a 2-fold increase in GROK conductance showed G_max_ had no appreciable impact on K^+^ flux at the free-running voltage nor did it accelerate stomatal kinetics. This conclusion is consistent with experiments overexpressing native (Wang et al. 2014a, 2014b; Nguyen et al. 2023) and optogenetic K^+^ channels (Papanatsiou et al. 2019) in vivo. Thus, introducing this new K^+^ conductance was expected to be sufficient to speed stomatal kinetics relative to the native GORK channel in *Arabidopsis*.

To test these predictions, we generated GROK-GFP and GORK-GFP constructs and stably transformed the *Arabidopsis gork* null mutant (Ache et al. 2000; Hosy et al. 2003) by floral dip (Clough and Bent 1998). We used the guard cell-specific promoter, pGC1 (Yang et al. 2008) with parallel complementation by the wild-type channel as a control. In general, plant channel promoters are not strong enough to give measurable signals under confocal microscopy [cf. (Homann and Thiel 2002; Meckel et al. 2004; Honsbein et al. 2009)], precluding a visual assessment of expression on a cell-by-cell basis. Furthermore, all evidence to date indicates that, alone, overexpressing plant K^+^ channels in vivo has no substantive effect on K^+^ flux [cf. (Honsbein et al. 2009; Wang et al. 2014a, 2014b; Horaruang et al. 2022); and above]. Transformants were selected for growth on Basta and for GFP fluorescence under confocal microscopy. We worked with the T1 and T2 generations as expression of the GORK channel is strongly suppressed in later generations, both with constitutive and the native promoters (Eisenach et al. 2014; Horaruang et al. 2022), and we anticipated the same issues with GROK. Thus, each plant represented an independent transformant and was carried through physiological and expression analyses.

**Figure 3.**
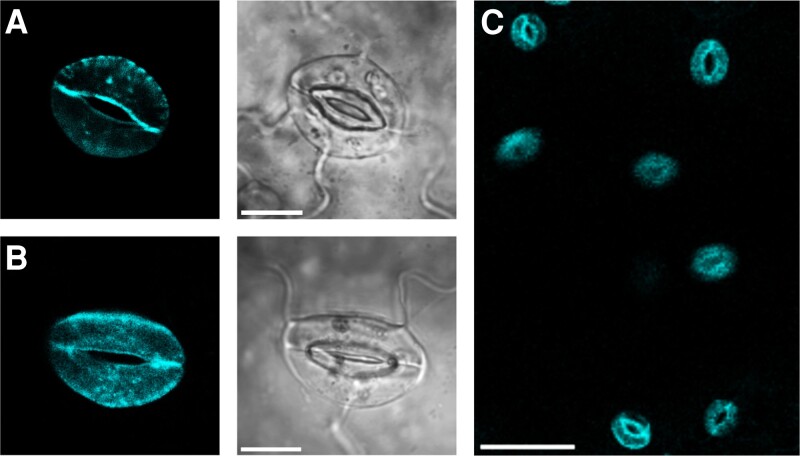
GROK-GFP localizes to the guard cells in stably-transformed *gork* mutant *Arabidopsis*. Confocal laser scanning microscopy (CLSM) images from the *Arabidopsis gork* mutant complemented with *Gynandropsis gynandra* GROK-GFP showing a peripheral punctate distribution similar to *Arabidopsis* GORK (Eisenach et al. 2014). **A** and **B)** Image projections of 2 representative guard cells with corresponding brightfield images. Scale bars: 10 µm. **C)** Lower magnification shows expression isolated to the guard cells. Scale bar: 50 µm.

**Figure 4.**
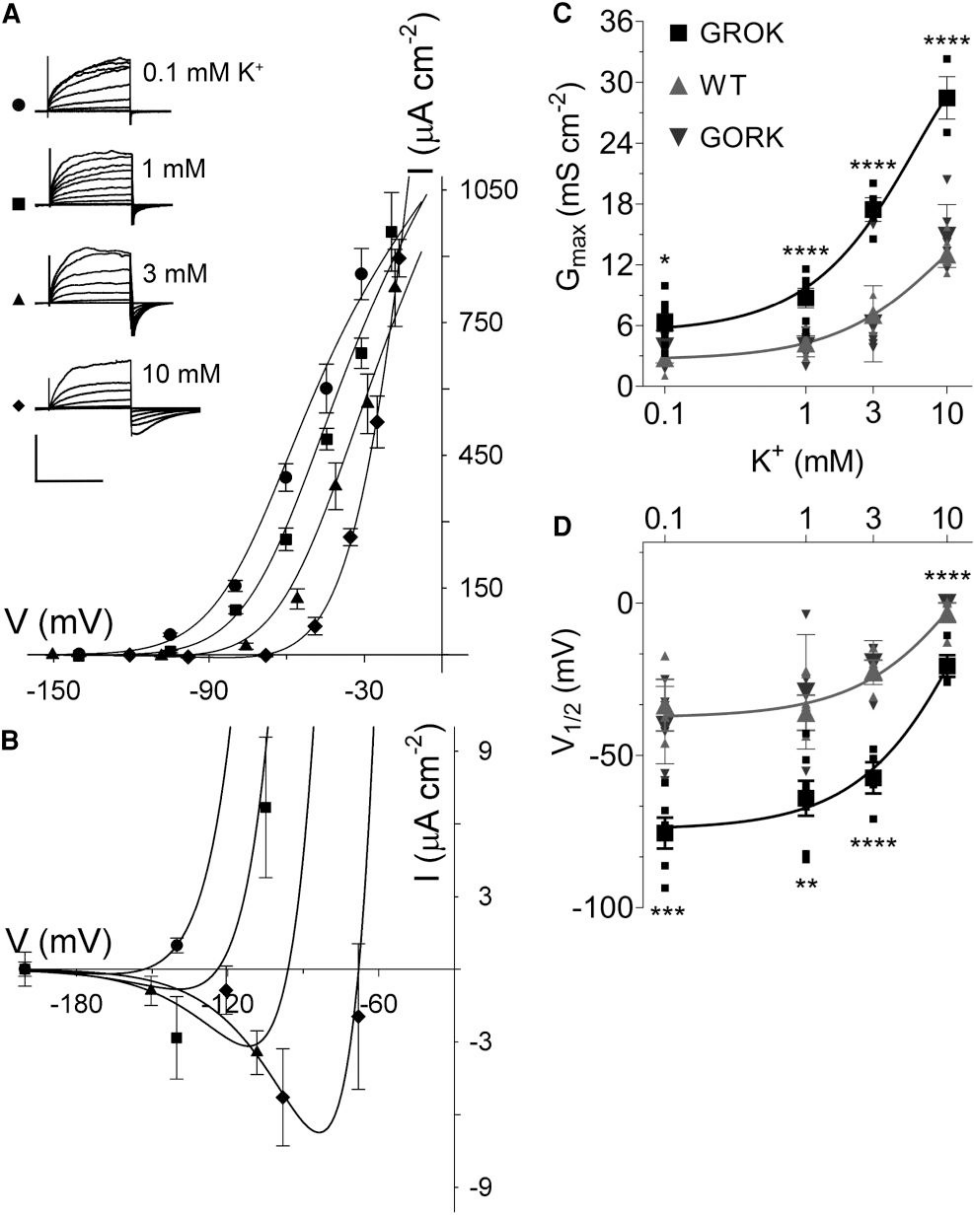
Guard cells of *GROK-*complemented *gork Arabidopsis* show a current that gates at voltages negative of those of wild-type *Arabidopsis*. **A)** Steady-state current-voltage (IV) curves recorded under voltage clamp from guard cells of the *Arabidopsis gork* mutant complemented with GROK-GFP. Data are means ± SE of 5 independent experiments. Guard cells were superfused with 5 mm Ca^2+^-MES, pH 6.1, and 0.1, 1, 3, and 10 mm KCl. Conditioning voltages, chosen outside the range of channel activity, were −200 mV (0.1 and 1 mm), −180 mV (3 mm), and −150 mV (10 mm KCl). Voltage steps ranged from −150 or −120 mV to −20 mV and were followed by a voltage step to the conditioning voltage. Steady-state IV curves were calculated from currents recorded at the end of the test steps after subtracting the instantaneous background at the beginning of each test step. Curves are joint, non-linear least-squares fittings to the Boltzmann function (Eqn [1]) using a Marquardt–Levenberg algorithm and E_K_ of −70 mV. Fittings yielded a common δ of 1.8 ± 0.1. *Inset*: Representative current traces cross-referenced by symbol. Scale bar: 10 s (horizontal), 900 μA/cm^2^ (vertical). **B)** Expanded view of the steady-state currents in **A)** shows an appreciable inward current carried by GROK at voltages negative of E_K_. For a typical *Arabidopsis* guard cell, a current of 1 μA cm^−2^ equates to a K^+^ flux of 80 Amol s^−2^ or roughly 8 mm min^−1^ on a cell volume basis. **C** and **D)** Maximum conductance (G_max_) and conductance midpoint voltages (V_1/2_) from fitting of the data in **A)** and from wild-type *Arabidopsis* (Alvim et al. 2024) and *GORK*-complemented *gork* mutant guard cells as a function of external K^+^ concentration. Means ± SE shown as larger symbols and individual data points as smaller symbols. Lines are first-order exponential functions and are included as a visual guide only. Asterisks indicate significant differences (*P ≤ 0.05, **P ≤ 0.01, ***P ≤ 0.001, ****P ≤ 0.0001) between lines after post hoc Tukey test.

As expected, we found that GROK-GFP localized to the guard cells and was distributed primarily to the cell periphery of the complemented *gork* mutants (Fig. 3). While we could not discount some internal labelling, electrophysiological analysis confirmed a substantial presence of the channel at the plasma membrane. On impalement and voltage clamping (Eisenach et al. 2014; Wang et al. 2017; Jezek et al. 2021; Horaruang et al. 2022), we recovered the characteristic GROK current (Fig. 4) exhibiting both a negative shift in V_1/2_ and greater G_max_ relative to wild-type *Arabidopsis* and the GORK-complemented *gork* mutant, much as observed in *Gyandropsis* guard cells (Alvim et al. 2024) and on GROK expression in oocytes (Fig. 2). Again, increasing external potassium concentration displaced the steady-state IV curves to more positive voltages (Fig. 4A). We also uncovered an appreciable inward current near and negative of E_K_ (Fig. 4B), sufficient in 1 mm K^+^ outside to add 8–18 mm min^−1^ to the rate of K^+^ uptake at voltages 10 to 20 mV negative of E_K_.

Of course, the actual rates of K^+^ uptake will depend on the prevailing membrane voltage, which is likely to vary, minute by minute, depending on the balance of the sum of ion movements across the membrane (Chen et al. 2012; Hills et al. 2012; Blatt 2024), across a range of voltages both positive and negative of E_K_ (Thiel et al. 1992; Konrad and Hedrich 2008; Eisenach et al. 2012; Wang et al. 2012; Blatt and Armstrong 1993; Blatt and Thiel 1994; Roelfsema et al. 2004). Tail currents in 10 mm K^+^ from *gork* null mutant *Arabidopsis* guard cells expressing GORK and GROK, respectively, yielded values for E_K_ of −73 ± 4 mV and −71 ± 3 mV and mean free-running voltages of −61 ± 4 and −65 ± 3 mV, the voltages of the GROK-transformed lines varying between −58 and −82 mV (Supplementary Fig. S6). So, the flux estimates most likely set an upper limit on the added K^+^ uptake evident on expressing GROK.

Even so, such additional rates of K^+^ flux would be sufficient to speed K^+^ accumulation by as much as 1.6-fold. Joint fittings to Eqn [1] yielded G_max_ values roughly 2-fold greater (Fig. 4C) than for GORK in wild-type *Arabidopsis* (Eisenach et al. 2014; Wang et al. 2017; Jezek et al. 2021; Horaruang et al. 2022); equally, V_1/2_ values were significantly more negative (Fig. 4D) than for GORK, at 1 mm K^+^ by −33 mV (Eisenach et al. 2014; Wang et al. 2017; Jezek et al. 2021; Horaruang et al. 2022). This difference was equivalent (Eqns [2,3]) to a decrease in the free energy for gating, ΔΔG, of −0.73 Kcal/mol and a decrease in affinity for K^+^ inhibition of over 2-fold.

### Gas exchange and growth of GROK-expressing Arabidopsis

**Figure 5.**
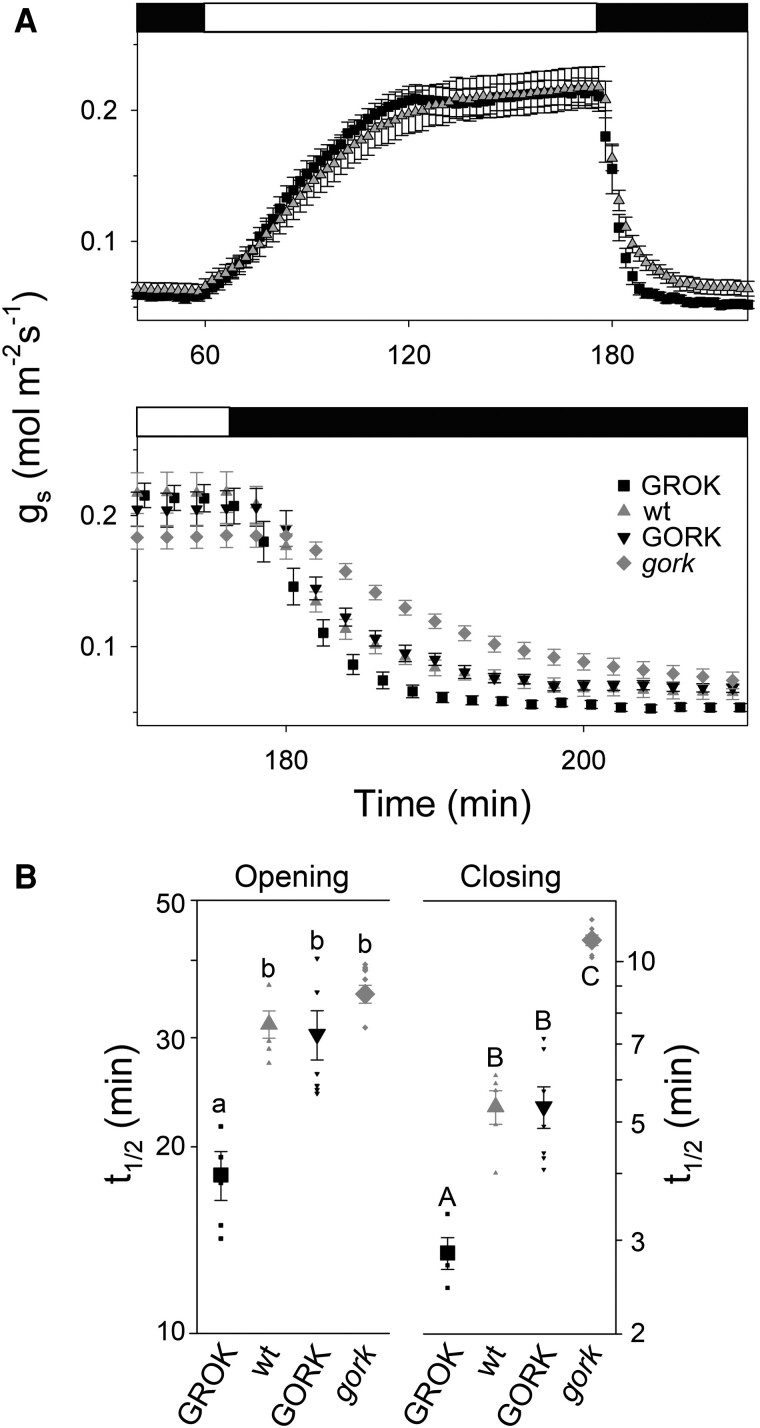
GROK complementation yields faster stomata. **A)** Stomatal conductance, g_s_, with steps between dark and 400 µmol m^−2^s^−1^ white light (bars above). Upper, Shows means ± SE for wild-type (gray triangles, *n* = 8) and *GROK-GFP*-complemented *gork* mutant Arabidopsis (black squares, *n* = 5). Data shown are every other data point and data for the *gork* mutant and *GORK-GFP*-complemented *gork* mutant *Arabidopsis* omitted for clarity. Lower, Shows these data on an expanded scale for the transition from light to dark and includes corresponding data for the *gork* mutant (gray diamonds, *n* = 8) and *GORK-GFP*-complemented *gork* mutant *Arabidopsis* (black triangles, *n* = 6). Data are means ± SE with symbols as indicated in the lower panel. **B)** Halftimes (t_1/2_) for opening and closing from data used to construct the means in **A)**. Individual data sets were subjected to non-linear least-squares fittings with single exponential functions. Small symbols are results of each fitting and large symbols are means ± SE for the genotype. Symbols as in **A)**. Letters indicate significant differences after post hoc Tukey test (*P* < 0.02).

We assessed the gas exchange characteristics of intact leaves of the same *GROK*-complemented *gork* mutant *Arabidopsis* and compared the results with the wild-type and *GORK*-complemented plants. Although stomatal conductances (g_s_) in the steady state were marginally lower in the *GROK*-complemented plants than those of wild-type and *GORK*-complemented plants, the differences were not significant. No differences were evident in stomatal size or density when comparing the *GROK*-complemented plants with either the Arabidopsis wild-type, or *Arabidopsis gork* mutant (Supplementary Fig. S7). We also observed no significant differences between the plant lines in photosynthetic capacity, as determined from measurements of steady-state carbon assimilation rates (*A*) in each case (Supplementary Fig. S7D). However, fitted g_s_ relaxations on opening and closing to a first-order exponential function showed that stomata of the *GROK*-complemented plants were significantly quicker in response, opening with halftimes 1.5-fold faster and closing with halftimes 2-fold faster than wild-type *Arabidopsis* (Fig. 5). Complementary results, albeit less pronounced, were obtained with steps in the partial pressure of CO_2_ (Supplementary Fig. S8). These differences, and those of the voltage clamp data, were not well-correlated with expression (Supplementary Fig. S9), suggesting that the effects could be ascribed to differences in channel gating. Thus, the faster opening and closing could be explained by the introduction of additional steady-state K^+^ flux capacity directed both outward and inward, as predicted in OnGuard simulations.

In the field, light can vary greatly over short time periods, for example as clouds pass overhead. Photosynthesis generally tracks the light energy, but stomata are much slower to respond. This hysteresis in stomatal kinetics can limit photosynthesis when the light increases and leads to water loss without commensurate assimilation when the light falls quickly (Pearcy 1990; Lawson and Blatt 2014; Long et al. 2022). We anticipated that the faster stomata of the *GROK*-complemented *Arabidopsis* might prove advantageous when daylight fluctuated. Therefore, we grew plants of all 4 genotypes on a 9:15 h day:night cycle, both under constant daylight and under light steps at 15-min intervals with the same total daily photon flux (Horaruang et al. 2022). With both light regimes, plants were divided between those grown water-replete and those grown with water limitation; soil water content was monitored therefore to maintain the soil moisture at 70 ± 5% and 10 ± 5%, respectively. After harvesting, plants were analyzed for rosette area, wet and dry biomass, and for K^+^ content as before (Papanatsiou et al. 2019; Horaruang et al. 2022).

**Figure 6.**
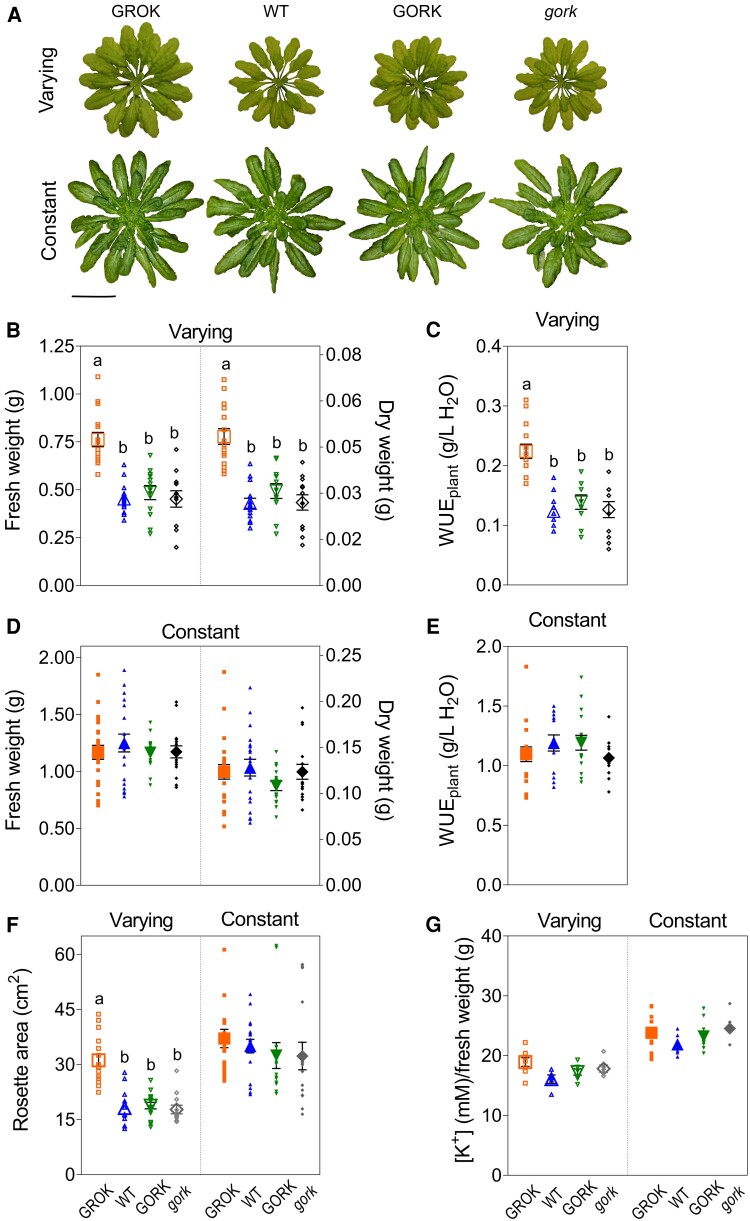
GROK enhances *Arabidopsis* biomass yield. **A)** Representative rosettes of the *GROK*-complemented *gork* mutant, wild-type *Arabidopsis thaliana*, *GORK*-complemented *gork* mutant, and the null *gork* mutant of *A. thaliana* grown under varying light and constant light regimes with soil water maintained at 10 ± 5%. Plants were harvested after 8 (constant light) and 10 weeks (varying light). Rosette images were digitally extracted from the background. Scale bar: 3 cm. Fresh weight, dry weight, and water use efficiency at whole plant level dry weight under **B** and **C)** varying (open symbols) or **D** and **E)** constant light (filled symbols) regimes. **F)** Rosette area and **G)** rosette potassium content under varying (open symbols) or constant light regime (filled symbols). Data (*n* > 10 for each genotype) are means ± SE. Small symbols are results of each fitting, and large symbols are means ± SE for the genotype. Letters indicate significant differences (*P* < 0.05) following post hoc Tukey test; no significant differences where lettering is absent.

We found that the *GROK*-complemented plants showed larger rosette areas, with fresh weights and dry biomass greater than any of the wild-type, *gork* mutant, and *GORK*-complemented *Arabidopsis* under varying light and especially when water-limited. All 4 genotypes showed slower growth, reduced rosette areas and biomass under the varying light regime when compared with constant daylight and were therefore grown for an additional 14 days (Fig. 6). However, the rosettes of *GROK*-complemented plants were larger and yielded biomass values of 1.6- to 1.9-fold greater when compared with any of the other genotypes within the same growth set (Fig. 6, B and C,F). By contrast, no differences were observed between the different lines when grown under constant light (Fig. 6, D and E,F). Again, the impact of GROK was not correlated with any differences in expression level (Supplementary Fig. S9).

As a measure of plant productivity, water use efficiency (WUE) defines the overall efficiency of dry mass production as a function of the water transpired. WUE is affected by light through the combined influence on carbon demand and associated transpiration (Pearcy 1990). We, therefore, calculated WUE for each plant as the dry aerial biomass divided by the total water supplied during the growth period (Halbritter et al. 2020). As expected, WUE of the *GROK*-complemented *Arabidopsis* was enhanced under water limitation compared with the other lines even though plant biomass was reduced under water limitation in each case (Fig. 6C). Again, no differences were observed under constant daylight (Fig. 6E). Similar, if less pronounced, differences were evident with water-replete growth (Supplementary Fig. S10). This increase in biomass was not the consequence of alterations in photosynthetic capacity per se (Supplementary Fig. S7D). It also did not reflect any differences in specific K^+^ content of the plants (Fig. 6G) that might have been associated with K^+^ channel expression (Horaruang et al. 2022). Thus, we conclude that expressing the GROK channel in *Arabidopsis*, and the accelerated stomatal kinetics it engenders, is responsible for the gains in biomass and WUE.

## Discussion

Manipulating the K^+^ conductance of guard cells to speed stomata has proven a viable strategy to reduce the time-averaged transpiration from leaves while promoting photosynthetic carbon capture (Papanatsiou et al. 2019; Horaruang et al. 2022). With the identity of the *Gyandropsis* guard cell K^+^ channel GROK, we can now show that the gating properties necessary and sufficient to speed stomatal movements have evolved naturally. Indeed, engineering K^+^ channel gating (Horaruang et al. 2022), in particular, was found previously to circumvent the implicit cost to photosynthetic carbon capture of reducing stomatal density (Lawson and Blatt 2014; McAusland et al. 2016; Caine et al. 2019). Our findings thus highlight the importance of guard cell membrane transport to the biology of C_4_ photosynthesis, opening the way for biological ‘mining’ of similar traits and comparative studies to resolve their structural and functional origins. By the same token, the findings will inform work towards the bioengineering of K^+^ flux and stomatal behaviours going forward.

To date, research to enhance yields in C_3_ plants by introducing characteristics of C_4_ photosynthesis had focused primarily on the anatomical and biosynthetic pathways needed to support a 4-carbon shuttle; these efforts include reconstructing the C_4_ Kranz anatomy and targeting and expressing key enzymes for (de)carboxylation in the vascular mesophyll (Giuliani et al. 2019; Ermakova et al. 2020, 2021; Kandoi et al. 2022). Our finding of the *Gynandropsis* GROK K^+^ channel—a close homologue of the *Arabidopsis* guard cell K^+^ channel GORK—underscores the importance of evolutionary changes to stomata that are needed to support C_4_ biology. Stomatal guard cells of C_4_ plants commonly are faster to respond to environmental factors affecting pC_i_ (Sage and Pearcy 2000; Osborne and Sack 2012; Ozeki et al. 2022).

While there is a wealth of information about guard cells of several C_3_ species (Jezek and Blatt 2017; Blatt 2024), what determines the mechanics of guard cells in C_4_ plants remains virtually unexplored. Beyond the obvious anatomical differences of stomata in C_4_ grasses, only in maize is there any quantitative information on ion transport in the guard cells (Fairley-Grenot and Assmann 1992; Mumm et al. 2011; Chen et al. 2017). These studies suggest some differences in cell-specific Ca^2+^ dependencies and regulation that may contribute to the shuttle of solute between the subsidiary and guard cells. However, the extant data offer few other insights. Thus, our discovery of the unique features of GROK gating, its equivalence to the native *Gynandropsis* K^+^ current (Alvim 2022), and its impact on stomatal kinetics in *Arabidopsis* (Figs. 2–4), points to stomatal speed native to the C_4_ plant that arises with GROK channel gating. Most important, these differences are clearly intrinsic to the channel protein itself.

### Channel gating and stomatal kinetics

Of the physiological features associated with GROK, the voltage- and K ^+^ -dependence of its gating stand out. Most significant, by virtue of displacing V_1/2_ to more negative voltages relative to GORK (Fig. 4), GROK gating enhanced the steady-state current at voltages positive of E_K_, thereby promoting steady-state K^+^ flux when the membrane depolarized for net solute loss. Similarly, this shift in V_1/2_ also introduced an appreciable inward current at voltages near and negative of E_K_ that added to the overall conductance for K^+^ influx at voltages typical of guard cells during stomatal opening (Thiel et al. 1992; Wang et al. 2012).

Expressing GROK yielded current amplitudes that were roughly 2-fold greater than those observed wild-type Arabidopsis and in plants expressing GORK under the same promoter [Fig. 4; see also Eisenach et al. (2014) and Horaruang et al. (2022)]. So, a cursory assessment might lead to a conclusion that the increase in G_max_ accounts for the enhanced K^+^ efflux and, hence, the faster stomatal kinetics. This interpretation fails on at least 3 accounts. First, because the conductance balancing a scalar—so, voltage-independent—increase in channel current is relatively constant, the increase in K^+^ conductance will be offset by an opposing shift in free-running membrane voltage that largely eliminates any gain in K^+^ efflux (Nguyen et al. 2023; Blatt 2024). This impact of voltage is amply documented both in quantitative simulation and in vivo experimentation (Wang et al. 2014a; Blatt et al. 2022; Horaruang et al. 2022).

Second, comparing expression as GFP fluorescence against channel gating and stomatal kinetic parameters failed to show any trend associating expression level with stomatal kinetics or channel gating per se (Supplementary Fig. S9). For example, both for GORK and GROK, the ensemble channel conductance (G_max_) showed no correlation to expression, at least over the range of fluorescence signals exhibited by the independent transformants. This finding is consistent with past studies that have shown weak correlations above a threshold of expression (Very et al. 1994, 1995).

It might be argued that overexpressing channels will lead to significant labelling of endomembrane compartments, thereby distorting the measure of expression. Certainly, the larger variations in G_max_ for GROK could be ascribed to differences in transcription, translation and post-translational activities, including the delivery of channels to the plasma membrane (Homann and Thiel 2002; Hurst et al. 2004; Meckel et al. 2005; Eisenach et al. 2014). However, using G_max_, as a measure of expression at the plasma membrane failed to show a correlation between expression and the key gating parameter V_1/2_ (Supplementary Fig. S9). So, expression, measured either as GFP fluorescence or as G_max_, clearly does not explain the differences in channel gating nor in the rates of stomatal opening and closing between the *GORK* and *GROK* complementation lines.

Third, quantitative simulations using the mechanistic OnGuard platform shows that varying G_max_ by 2- to 3-fold has little or no impact on K^+^ flux and the rates of stomatal movements, whereas altering the V_1/2_ for K^+^ channel gating, even by ±18 mV does (Wang et al. 2014a; Horaruang et al. 2022; Nguyen et al. 2023). Again, these predictions find validation in experiments overexpressing 2 related K^+^ channels (Wang et al. 2014b).

How, then, can we understand this difference in effects? By contrast with a scalar increase in channel conductance, altering the voltage dependence of K^+^ channel gating has the effect of “repositioning”, with respect to voltage, the K^+^ conductance relative to the conductances that must balance K^+^ movement. Provided that this repositioning also favors the balancing conductance, the effect is to promote efflux of both K^+^ and its counter charge, regardless of the membrane voltage. The situation is even more straightforward for K^+^ influx: in this case, the respositioned gating introduces a significant conductance for K^+^ at voltages negative of E_K_ [Fig. 4; see also Horaruang et al. (2022)], so promoting K^+^ influx when H ^+^ -ATPase activity drives the membrane across these voltages. In other words, it is the altered voltage dependence for K^+^ flux that is essential to enhancing the rates of K^+^ uptake during opening and its loss during stomatal closure. Again, these are conclusions that find validation both through quantitative simulation and in vivo experimentation (Papanatsiou et al. 2019; Horaruang et al. 2022).

Finally, we note that GROK showed somewhat faster gating kinetics, at least at voltages promoting channel opening and when expressed in oocytes [Fig. 2; tail currents in vivo are often slower to relax, which may reflect the native environment (Eisenach et al. 2014; Grefen et al. 2015; Horaruang et al. 2022)]. However, it is important not to confuse net steady-state K^+^ flux with the kinetics of channel gating on a voltage step. Stomatal movements depend on the cumulative flux of K^+^ passing through the channels over many minutes under a largely stable (steady-state) voltage, not on how fast or slow the channels open and close under voltage clamp. Current kinetics on a step in clamp voltage informs an understanding of the mechanism of channel gating, but they are not relevant to steady-state K^+^ flux or stomatal kinetics. Thus, in summary, the acceleration in stomatal kinetics, both on closing and on opening, is most easily understood to arise from the shift in V_1/2_ intrinsic to GROK and its impact on steady-state K^+^ flux.

### The determinants of GROK K^+^ dependence

An open question now is whether similar physiological characteristics in other C_4_ species may be linked to the properties of the homologous channels. Given the high conservation of the GROK channel sequence with its counterpart in *Arabidopsis* (Supplementary Figs. S2 and S3), what structural characteristics determine its gating properties remain a puzzle. Analysis of the *Arabidopsis* GORK channel identified the N-terminal clustering domain as a structural element that facilitates K ^+^ -dependent inhibition of the channel gate (Horaruang et al. 2022); mutations eliminating GORK channel clustering, through alterations in alternately charged subdomains, displaced channel gating to more negative voltages, promoting K^+^ flux and stomatal speed, and enhancing WUE and biomass gains. However, the study also indicated the presence of sites internal to the 3-dimensional structure of GORK that were proposed to be necessary for K^+^ inhibition of the gate, and it did not address contributions from the most N-terminal sequences of residues. Both regions are likely to be equally important for channel gating and its K^+^ inhibition.

Even so, alignments of GROK with GORK and other outward-rectifying K^+^ channels do not uncover any outstanding differences in charge distributions within the N-terminal clustering domain that might account for the relaxed K^+^ inhibition of the GROK channel (Supplementary Fig. S3). Thus, it is possible that the characteristics of GROK are linked to internal residue differences, possibly those altering intra-subunit binding affinities, much as was inferred from the GORK mutants (Horaruang et al. 2022). Until the crystal structure of GORK is resolved, we can only guess where these sites reside from homology models based on other plant CNBD-type channels (Clark et al. 2020; Dickinson et al. 2022; Li et al. 2023).

It is of interest that we observed little difference in weight or WUE between the wild-type and *gork* null mutant, notably under varying light (cf. Figure 6, A to C). There are several possible explanations for a lack of impact of the null mutant, not least that against a background of other conductances the K^+^ flux through GORK may account for only a 20% to 30% difference in transpiration compared to wild-type Arabidopsis (Hosy et al. 2003). We note, too, that the 15-min steps in light were chosen to reveal the effects of GROK relative to the wild-type control; however, the period of these steps was sufficiently short that differences between wild-type and *gork* mutant plants will have been masked. This latter explanation accords with the halftime midpoints for closing and opening (Fig. 5B) that define the differences in response characteristics between the plant lines. For GROK, this midpoint was close to 11 min whereas, for the wild-type and *gork* mutant, the values were 19 and 26 min, respectively, substantially greater than the 15-min switching window for stomatal response. So, for the wild-type and *gork* mutant plants, these values imply substantial damping in the stomatal frequency-response characteristics that will have masked differences between the *gork* mutant and wild-type plants by comparison with the GROK-complemented plants within the windows of light and dark steps. In effect, the slower response characteristics of the *gork* mutant and wild-type control provided low-pass filtering that minimized their differences.

Finally, our findings underscore the importance of channel gating to manipulating membrane transport. Much as we find in simulations with GROK, systems analyses of stomata (Wang et al. 2014a; Jezek et al. 2021; Horaruang et al. 2022) had shown previously that altering channel populations alone has only a marginal impact on stomatal performance and that targeting the control of transport, including channel gating, is most effective. This conclusion accords with trials manipulating pump and channel populations (Kusumi et al. 2012; Wang et al. 2014a, 2014b; Toh et al. 2021) that generally have shown conflicting actions on WUE and biomass gain. Our finding of a naturally-occurring K^+^ channel that speeds stomatal movements underscores the importance of gating native to the channel in accelerating stomatal kinetics to match the needs for guard cell ion flux under a changing light environment (McAusland et al. 2016). Thus, the findings show how stomatal speed is a natural and effective strategy to circumvent the often opposing demands behind safeguarding water use, thereby gaining in photosynthetic assimilation while reducing water demand during vegetative growth.

## Materials and methods

### Plant growth

Wild-type, mutant and transformed *A. thaliana* Col0 were grown under 9:15 h L:D, or for floral dipping under 15:9 h L:D, with 140 *μ*mol m^–2^s^–1^ PAR at 22:18 °C and 60:70% relative humidity (RH). Tobacco (*N. tabacum*) was grown under 15:9 h L:D with 200 μmol m^−2^s^−1^ PAR at 26:22 °C and 60:70% relative humidity (RH). Stable transformants of the *Arabidopsis gork* mutant were generated by floral dip (Clough and Bent 1998) and were selected for GFP fluorescence by confocal microscopy after growth with 35 μM Basta. Transient transformation of tobacco was by infiltration with *Agrobacterium tumifaciens* as described previously (Eisenach et al. 2014). For experiments with varying light, plants were grown as described previously (Horaruang et al. 2022) under a 9:15 h L:D regime at 50%RH and 22:18 °C, either under constant daylight of 100 μmol m^−2^s^−1^ PAR or varying at 15-min intervals with a maximum of 200 μmol m^−2^s^−1^ to give the same total daily fluence.

### Molecular biology

*Gynandropsis gynandra* were germinated (Aubry et al. 2016) and grown for 7 days. RNA was extracted using RNeasy Plant Mini Kit (QIAGEN) from 120 mg of cotyledon tissue and verified by the OD_260_/OD_280_ ratio on a NanodropOne (Thermo Scientific) microspectrophotometer. cDNA was synthesized using a QuantiTect Reverse Transcription Kit (QIAGEN). The GROK channel (Gg13038) coding sequence was amplified without stop codon by PCR with forward and reverse primers GGGGACAAGTTTGTACAAAAAAGCAGGCTTAATGATGTGTCTGCTGCGGA and GGGGACCACTTTGTACAAGAAAGCTGGGTTGTATGTTTGATTTGTTGCGACCAAA, respectively, including the Gateway attB sites, using the *Gynandropsis* cDNA as a template. The PCR product was isolated, checked for size by agarose gel electrophoresis, excised and extracted before Gateway (Life Technologies, Paisley, UK) cloning. The Entry clone pDONR207Gg13038-wo was constructed by BP reaction with the pDONR207 Entry vector and transformed into *Escherichia coli* Top10 competent cells. Transformants were selected, and DNA sequences verified by sequencing before LR reactions to generate Destination clones (Supplementary Table S2).

For reverse transcription quantitative PCR (RT-qPCR), whole leaves and epidermal peels of *Gynandropsis* were used. Epidermal peels were collected by hand and adhering mesophyll removed before repeated washing cold distilled water. *Gynandropsis* does not have anatomically-distinct subsidiary cells and visual inspection showed a monolayer of living guard cells only, as before (Alvim et al. 2024). RNA was isolated using the RNeasy Plant Mini Kit (Qiagen), and cDNA from the samples was synthesized following QuantiTect Reverse Transcription Kit's (Qiagen) protocol. qPCR was performed using PowerTrack SYBR Green Master Mix (Thermo Fisher Scientific). Gene-speciﬁc primers were designed for Gg13038 (forward, CGGAGAGGATTCACAGACGG; reverse, TGCAGCGGTTGAGATGAGTT), Gg10030 (forward, CGCGTTGAAGGTGAATAGCG; reverse, AGAGATGCAAAGGCGACCTC), Gg22935 (forward, GTGGTAAAGGGCGACAGTGA; reverse, TTCCCCCATCTGTCCTTTGC); and Gg00000300 (GgACT2, forward, GCTGAACGTGAAATCGTCCG; reverse, ATGGCTGGAACAGCACCTC). Primers were used to detect expression in whole leaf and epidermal peels. Relative transcript levels were normalized to Actin gene 2 (Gg00300) in each sample using standard curves calculated for the individual PCR products.

### Confocal microscopy

Transformed leaf tissues were imaged using a Leica SP8 SMD confocal microscope (Leica, Wetzlar, Germany) with HC PL APO 20×/0.75 and HC PL APO 40×/1.30 oil lenses and hybrid GaAs detectors. Fluorescence was excited at 488 nm for GFP and 552 nm for RFP, and fluorescence emission was collected across 495 to 550 nm and 560 to 620 nm, respectively, using standardized settings. Chloroplast autofluorescence was excited with 488 nm and collected across 630 to 690 nm. Images were processed using ImageJ v. 2.30 (image.nih.gov/ij/).

To quantify channel expression in *GORK*- and *GROK*-transformed *Arabidopsis*, leaf sections were isolated and, before mounting, were vacuum-infused with water to eliminate air pockets that otherwise yield false fluorescent signals through light scattering (Fricker et al. 2006; Sutter et al. 2006). Z-stack projections were assembled prior to fluorescence counting and the mean fluorescence was determined from at least 20 projections from each plant to calculate the relative fluorescence signal for the plant. Because electrical recordings were necessarily made on guard cells in freshly-peeled epidermis from intact leaves, it was not possible to undertake recordings on the same guard cells that were imaged from leaf sections and these measurements were carried out on guard cells in epidermal peels from neighbouring leaves of the same plant. For the same reasons, gas exchange measurements were carried out on neighbouring leaves of the same plant.

### Electrophysiology

Linearized destination clones were transcribed in vitro using the mMESSAGE mMACHINE T7 kit (Ambion, Life Technologies, Paisley, UK) and cRNA quantified before dilution for *Xenopus* oocyte injections at 25 ng/oocyte, as described previously (Eisenach et al. 2014; Horaruang et al. 2022). Currents were recorded by 2-electrode voltage clamp under continuous superfusion with 10 mm HEPES-NaOH, pH 7.3 and 1, 3, 10, and 30 mm KCl, with osmolarity adjusted using mannitol. Oocytes expressing channels from each experiment were collected for analysis by immunoblot using anti-GFP (Chromotek) primary antibodies, as before (Eisenach et al. 2014; Horaruang et al. 2022). Recordings from *Arabidopsis* guard cells in epidermal peels were carried out by 2-electrode voltage clamp under continuous superfusion with 5 mm Ca^2+^-MES buffer, pH 6.1, and 0.1, 1, 3, and 10 mm KCl, as before (Eisenach et al. 2014; Horaruang et al. 2022). All voltage clamp and analysis used Henry's EP Suite (www.psrg.org.uk) and SigmaPlot v.11.2 (SPSS, Poole UK).

### Gas exchange, biomass and WUE analysis

Infra-red gas exchange measurements were carried out using Li-COR 6800 gas exchange systems (Lincoln, USA) equipped with a Multiphase Flash Fluorometer (6800-01A, LI-COR) as previously described (Papanatsiou et al. 2019; Jezek et al. 2021; Horaruang et al. 2022). For biomass and WUE analysis, plants were imaged and surface areas calculated using ImageJ. Aerial tissues were harvested, and fresh and dry weights were recorded for each plant. Dried matter was extracted in 2 m HCl for K^+^ analysis by flame photometry (Sherwood Scientific, Cambridge, UK) against known standards as before (Eisenach et al. 2014; Horaruang et al. 2022).

### Stomatal assays

Guard cell size and stomatal densities were recorded from fresh abaxial epidermal peels using AmScope Microscope Digital Camera (MU1803 USB3.0 18MP Colour) attached to a Zeiss Axiovert 35 microscope using PL APO 10×/0.3 NA and PL APO 20×/0.6 NA objectives. Measurements were of randomized images (*n* > 60) of at least 4 different plants for each line, quantified using ImageJ (image.nih.gov/ij/), and the data pooled for means ± SE.

### OnGuard3 modelling

OnGuard3 (v. 3.3.1.6) was built on the HoTSig platform (Chen et al. 2012; Hills et al. 2012; Wang et al. 2017; Jezek et al. 2021), incorporating water vapor and CO_2_ diffusion between the atmosphere and interior of the leaf as well as the visco-elastic characteristics of the cell wall and surrounding epidermis. OnGuard3 models were driven through a diurnal light:dark cycle as before with steps in light imposed on this cycle as indicated, and all model outputs were derived from this cycle. Constant apoplastic solute contents were defined, and primary, energy-dependent transport, sucrose and malic acid synthesis within the guard cell were coupled to light as before (Jezek et al. 2021). Light input also contributed to the rate of carbon fixation by the leaf mesophyll and, hence, to the sink for CO_2_ within the leaf according to established relationships between light, CO_2_ and carbon assimilation. All other model parameters were fixed. The properties of the individual transporters, metabolism and buffering reactions thus responded only to changes in model variables arising from the parameters encoded in the model (Supplementary Appendix 2). Note that OnGuard3 models will faithfully reproduce a given set of outputs time and again for any one set of parameters. Statistical analysis of these outputs is therefore meaningless. OnGuard3 and the models for wild-type, *gork*, and V_1/2_-shifted variant mGROK Arabidopsis are freely available for academic users for download from www.psrg.org.uk and www.plantscienceglasgow.org.

### Statistical analysis

Data are presented as means ± SE of *n* observations. Significance was determined by ANOVA and post hoc using Tukey or Students *t* test (2-tailed distribution and 2-sample equal variance) and is indicated at *P* < 0.05 unless otherwise indicated.

### Accession numbers

Sequence data from this article can be found in the GenBank/EMBL data libraries under accession numbers_listed in Supplementary Table S1.

## Supplementary Material

kiaf039_Supplementary_Data

## Data Availability

Correspondence and requests for materials should be addressed to M.R. Blatt (michael.blatt@glasgow.ac.uk). All data and materials included herein are available on reasonable request.

## Supplementary data

The following materials are available in the online version of this article.

**Supplementary Figure S1.** Phylogenetic relationship of voltage-gated K^+^ channels from selected species with those of *Gynandropsis gynandra*.

**Supplementary Figure S2.** Amino acid alignment of *Arabidopsis thaliana* GORK with the predicted sequence for *Gynandropsis gynandra* 13038.

**Supplementary Figure S3.** Conserved alternation of subdomain charges between GORK-like channels.

**Supplementary Figure S4.** OnGuard3 modelling predicts an enhanced capacity for K^+^ flux through the GROK channel

**Supplementary Figure S5.** OnGuard3 model output comparison highlights K^+^ flux through the mutant GORK channel

**Supplementary Figure S6.** Stomatal dimensions, stomatal density, and carbon assimilation of Arabidopsis genotypes.

**Supplementary Figure S7.** Stomata of GROK-complemented *Arabidopsis* respond rapidly to changes in atmospheric CO_2_.

**Supplementary Figure S8.** Expression levels for the GROK- and GORK-complemented Arabidopsis do not correlate with phyisology.

**Supplementary Figure S9.** GROK-complemented Arabidopsis show enhanced biomass yield under varying light when grown water-replete.

**Supplementary Table S1.** Amino acid sequence identity matrix for *Gynandropsis gynandra* channels with voltage-gated K^+^ channels of *Arabidopsis thaliana* and outward-rectifying K^+^ channels of *Tarenyana hassleriana*.

**Supplementary Table S2.** List of constructs.

**Supplementary Appendix 1.** Amino acids sequences of outwardly—rectifying K^+^ channels of *Gynandropsis gynandra* genes

**Supplementary Appendix 2.** OnGuard3 parameter set highlighting GORK and mGROK gating

## Dive Curated Terms

The following phenotypic, genotypic, and functional terms are of significance to the work described in this paper:

GORK Gramene: AT5G37500
GORK Araport: AT5G37500

SKOR Gramene: AT3G02850
SKOR Araport: AT3G02850

guard cell AmiGo: PO:0000293

SYP121 Gramene: AT3G11820
SYP121 Araport: AT3G11820

CLSM Gramene: confocal laser scanning microscopy
CLSM Araport: confocal laser scanning microscopy

## Dive Curated Terms

The following phenotypic, genotypic, and functional terms are of significance to the work described in this paper:

GORK Gramene: AT5G37500
GORK Araport: AT5G37500

SKOR Gramene: AT3G02850
SKOR Araport: AT3G02850

guard cell AmiGo: PO:0000293

SYP121 Gramene: AT3G11820
SYP121 Araport: AT3G11820

CLSM Gramene: confocal laser scanning microscopy
CLSM Araport: confocal laser scanning microscopy
