# Supplementary material for: A C4 plant K+ channel accelerates stomata to enhance C3 photosynthesis and water use efficiency
Source: Plant Physiol. 2025 Jan 24;197(2):kiaf039. doi: 10.1093/plphys/kiaf039 (PMC11837344; doi:10.1093/plphys/kiaf039)
Supplement: kiaf039_Supplementary_Data [file kiaf039_supplementary_data.pdf]

Supplemental Materials

Supplementary Figures

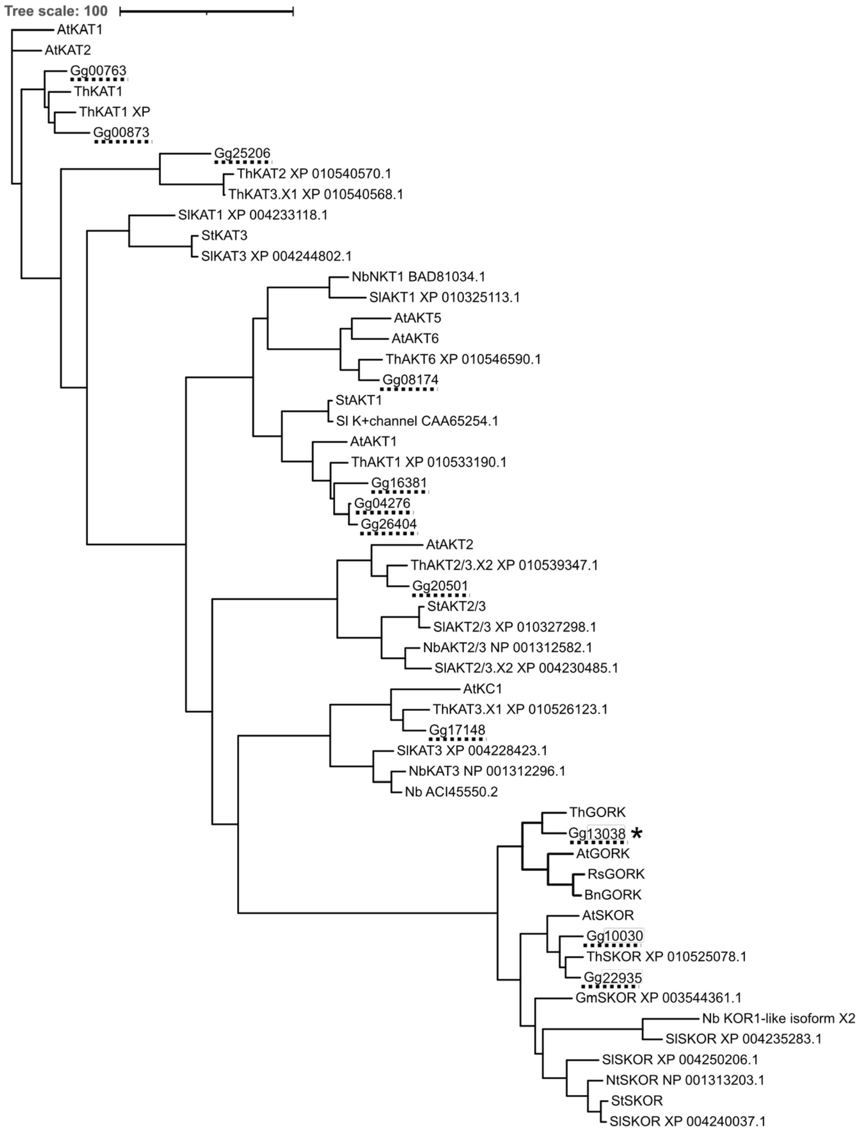

Supplementary Figure S1. Phylogenetic relationship of voltage-gated K^+ channels from selected species with those of *Gynandropsis gynandra*.

Phylogenetic analysis by maximum likelihood using BioEdit 7.2. Tree image constructed using online tool: https://itol.embl.de. Asterisks highlight the *Gynandropsis* 13038 gene product. Other putative *G. gynandra* K^+ channels are underlined (dotted lines). Abbreviations: At = *Arabidopsis thaliana,* Gg = *Gynandropsis gynandra*, Th = *Terenya hassleriana*; Sl = *Solanum lycopersicu* (tomato), St = *Solanum tuberosum* (Potato), Bn = *Brassica napus*, and Rs = *Raphanus sativus*. Tree scale, relative distance.

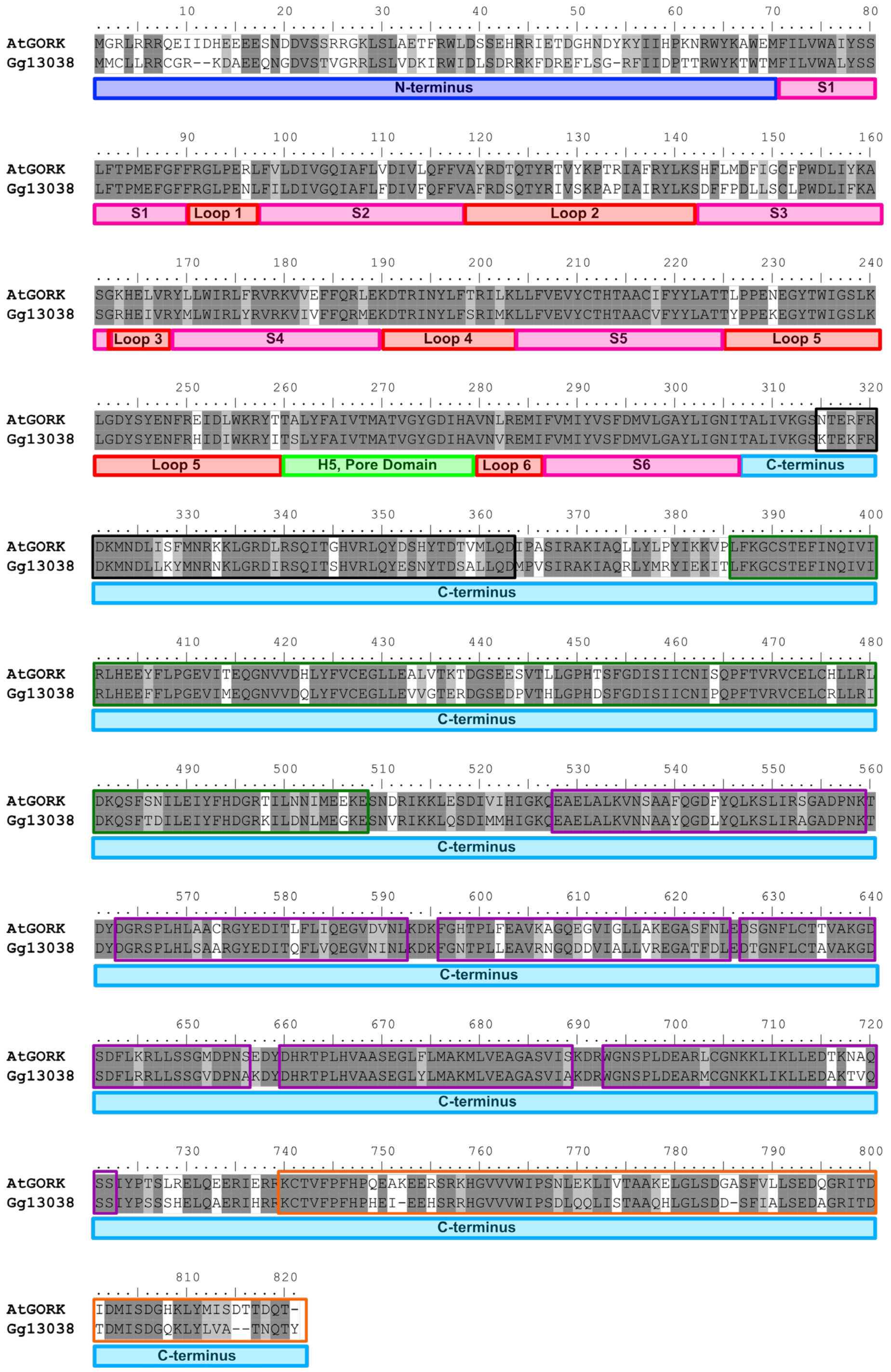

Supplementary Figure S2. Amino acid alignment of *Arabidopsis thaliana* GORK with the predicted sequence for *Gynandropsis gynandra* 13038.

Highlighting below indicates cytosolic N-terminus (dark blue), transmembrane domains S1 to S6 (pink), extracellular and cytosolic loops (red), extracellular pore domains (H5, green), and cytosolic C-terminus (light blue). The C-terminus contains a so-called C-linker domain (black box), a CNBD domain (dark green box), six ANK domains (purple boxes), and a KHA domain (orange box). Dark gray indicates amino acids that are identical (77%), light gray indicates

similarity (87%). Amino acids identical by channel segments are 36%, N-terminus; 84%, S1-S6; 78%, C-terminus. Residue numbering is to GORK.

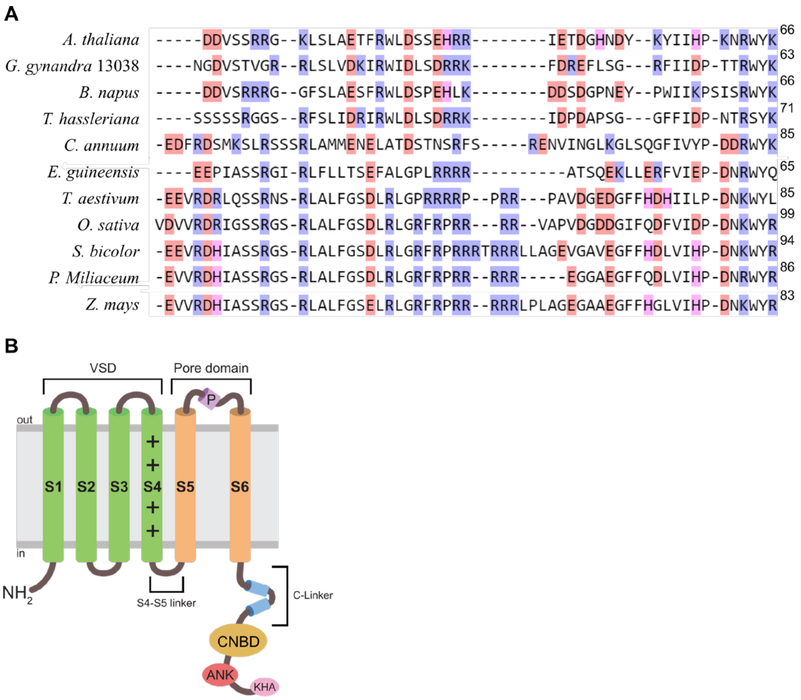

Supplementary Figure S3. Conserved alternation of subdomain charges between GORK-like channels.

(A) Alignment of the N-terminal cytosolic domains of GORK-like channels highlights an alternation of positively and negatively charged subdomains. Sequences (National Center for Biotechnology Information identifiers) from C_3 *Arabidopsis thaliana* GORK (CAC17380.1), the *Arabidopsis* relative C_4 *Gynandropsis gynandra* (13038), *Brassica napus* (XP_013656069.1), the C_3 *Arabidopsis* relative *Tarenaya hassleriana* (XP_010553885.1), and C_3 plants *Elaeis guineensis* (XP_010905454.2), *Capsicum annuum* (KAF3658364.1), *Triticum aestivum* (XP_044442180.1), and *Oriza sativa* (XP_015644419.1). Included are sequences for the C_4 plants *Sorghum vulgare* (XP_021305239.1), *Panicum miliaceum* (RLM54412.1), and *Zea mays* (PWZ19722.1). Charged residues (positive, blue; negative, red) are highlighted. **(B)** Structural schematic of a single polypeptide of the CNBD subfamily of voltage-gated K^+ channels. Functional channels assemble from four homologous polypeptides. Each polypeptide comprises a cytosolic N-terminus, transmembrane helices S1 to S4 (green) that form the voltage-sensor domain (VSD), the pore-lining helices S5 and S6 (Pore domain, orange) and pore-loop (P, lilac) that incorporates the K^+ channel TxGYGD motif. The cytosolic C-terminus contains a C-linker domain (blue), CNBD (cyclic nucleotide binding domain, dark

yellow), ankyrin (ANK, red), and KHA (hydrophobic and acidic residues, pink) domains. Positively charged amino acids in the S4 helix are also highlighted.

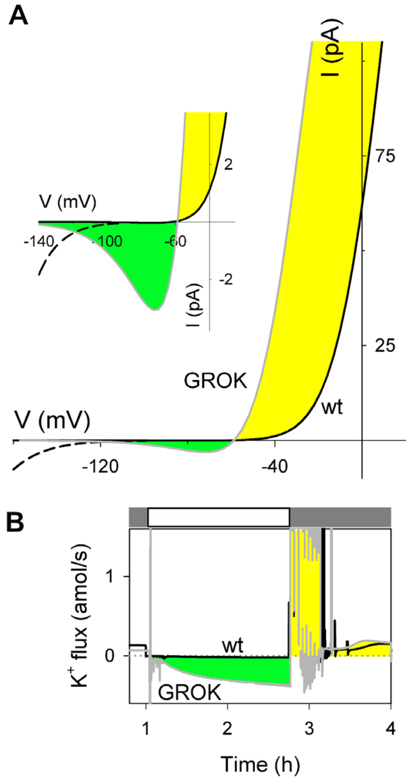

Supplementary Figure S4. OnGuard3 modelling predicts an enhanced capacity for K^+ flux through the GROK channel

(**A**) Model current-voltage (IV) curves for the wild-type (wt, black line) and the GORK channel mutated to displace the K^+-dependence of gating by -25 mV (GROK, grey line). IV curve for the KAT current (dashed line) included for reference. Curves correspond to the channel current with 10 mM K^+ outside. *Inset*: IV curves plotted on an expanded current scale. Shading indicates the enhanced capacities for K^+ efflux (yellow) and K^+ influx (green) at voltages positive and negative of E_K, respectively. (**B**) Model K^+ flux in the wild-type (wt, black line) and variant GROK (grey line) as in (A) over time with a light step from 10 to 200 μmol $m^{-2}s^{-1}$ PAR (*above*). Shading indicates the enhanced K^+ efflux (yellow) and K^+ influx (green).

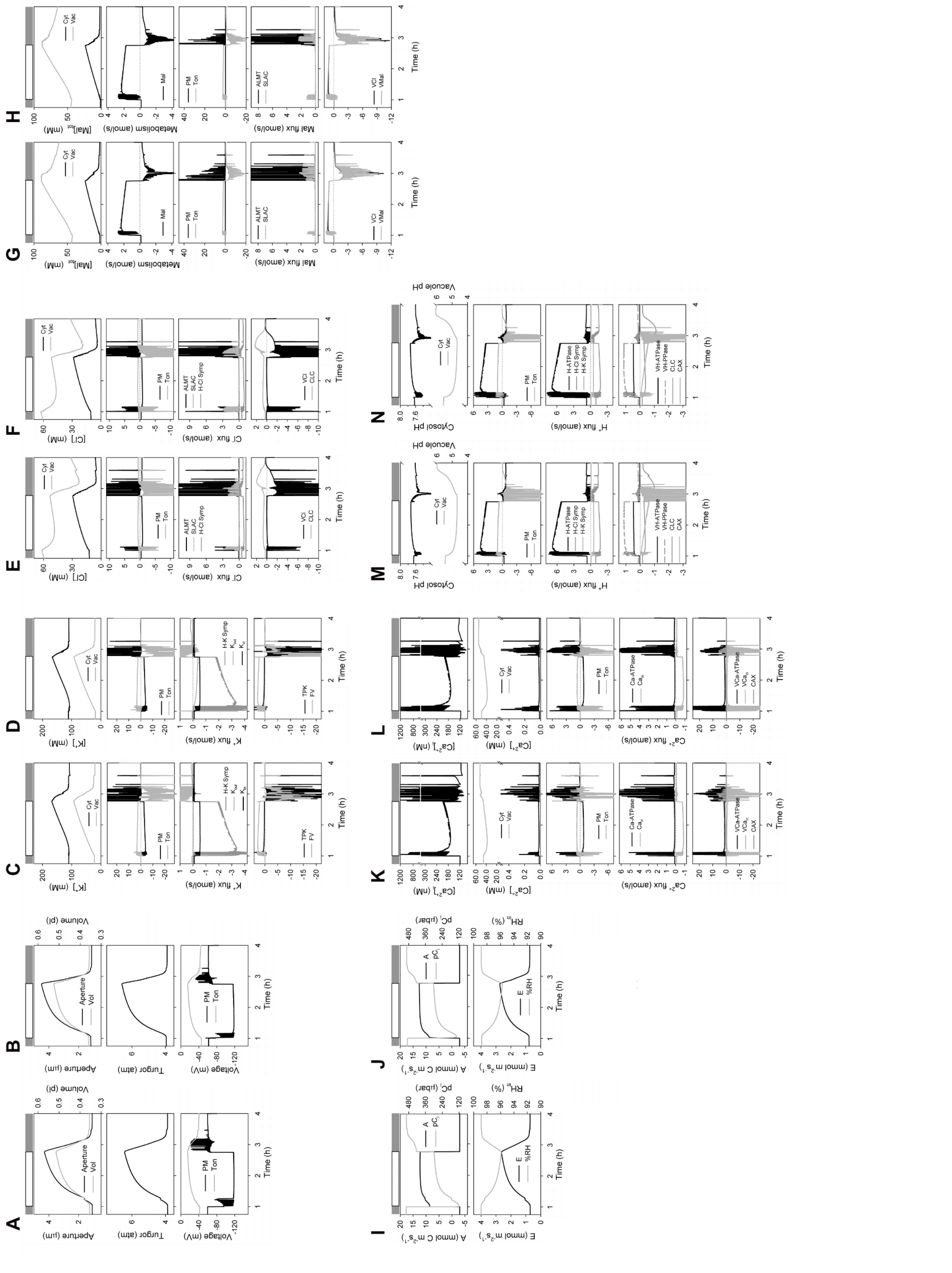

Supplementary Figure S5. OnGuard3 model output comparison highlights K^+ flux through the mutant GORK channel

Model outputs generated using a light step of 100 min to 200 mol $m^{-2}s^{-1}$ PAR following a 1-h period of 10 mol $m^{-2}s^{-1}$ PAR (*above*). Model parameters are listed in Appendix 1. Graphs are separated in column pairs for the wild-type (A, C, E, G, I, K and M) and with GROK gating (B, D, F, H, J, L and N) displaced by -30 mV (see Supplemental Fig. S5). Outputs are for (**A**,**B**) aperture, cell volume, turgor and voltage; (**C**,**D**) K^+ content and flux ; (**E**,**F**) Cl^- content and flux; (**G**,**H**) Mal content, synthesis and flux; (**I**,**J**) assimilation, transpiration, internal relative humidity (%RH) and stomatal conductance, g_s; (**K**,**L**) Ca^{2+} free concentration, total content and flux; and (**M**,**N**) pH and H^+ flux. An expanded view of the K^+ flux through GORK for the wild-type and GROK simulations is included in Supplemental Fig. S5. Note that positive flux is defined as movement of the ionic species (not charge) out of the cytosol, either across the plasma membrane or the tonoplast. Mal synthesis is indicated by positive values (G,H). For each ion, the total flux across the plasma membrane (PM) and tonoplast (Ton) is given with the individual flux components for the two membrane shown in the two panels below. Abbreviations for the individual transporters cross-reference to the list in Appendix 1. Oscillations in a number of fluxes are a consequence of the corresponding oscillations in voltage and cytosolic free [Ca^{2+}] ([Ca^{2+}]$_i$). A scan of the flux behaviours across the various solutes shows that only K^+ flux through the GORK and GROK channels (third panel down in c and d) differs appreciably between the two simulations.

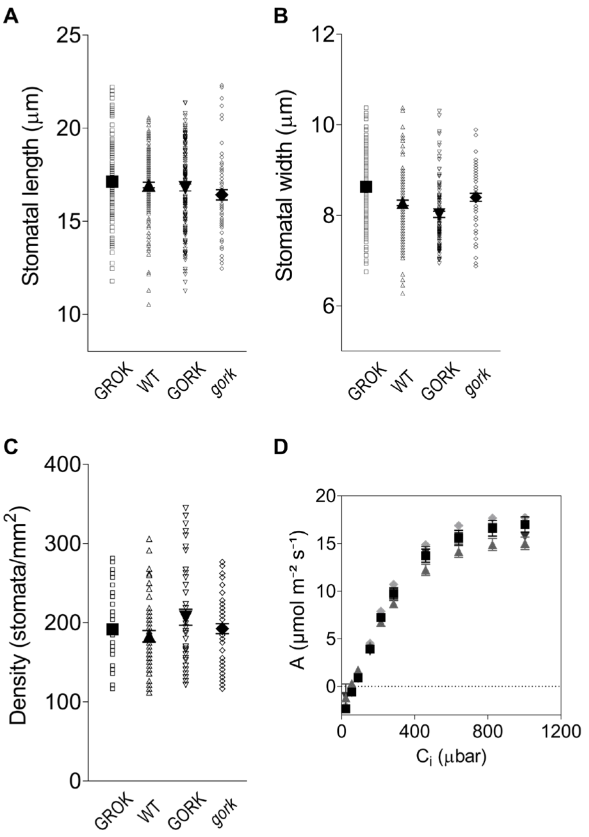

Supplementary Figure S6. Stomatal dimensions, stomatal density, and carbon assimilation of Arabidopsis genotypes.

(A, B) Stomatal dimensions, **(C)** density and carbon assimilation **(D)** of GROK-GFP complemented *gork* mutant Arabidopsis (squares), wild-type Arabidopsis (triangles), GORK-GFP complemented *gork* mutant Arabidopsis (inverted triangles) and the *gork* mutant (diamonds). Steady-state carbon assimilation (*A*) plotted as a function of the calculated C_i within the leaf under 600 µmol $m^{-2}s^{-1}$ of light. Data are means ±SE. No statistical differences were observed between the genotypes.

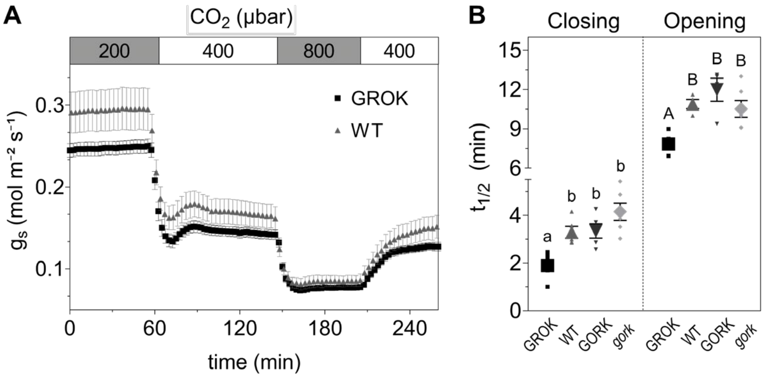

Supplementary Figure S7. Stomata of GROK-complemented *Arabidopsis* respond rapidly to changes in atmospheric CO_2.

(A) Stomatal conductance (g_s) recordings from GROK-complemented *gork* mutant (black squares, n = 7) and wild-type Arabidopsis (gray triangles, n = 5) with steps in external CO_2 (above). Data are means ±SE. Every other data point omitted for clarity. **(B)**: Halftimes ($t_{1/2}$) for responses to CO_2 steps in (A) includes corresponding data for the *gork* mutant and the GORK-complemented *gork* mutant. Kinetics were extracted by non-linear least-squares fittings to single exponential functions. Data shown for individual experiments (small symbols) and the corresponding means ±SE (larger symbols, n>6 for each genotype) with letters indicating significant differences after post-hoc *Tukey* test.

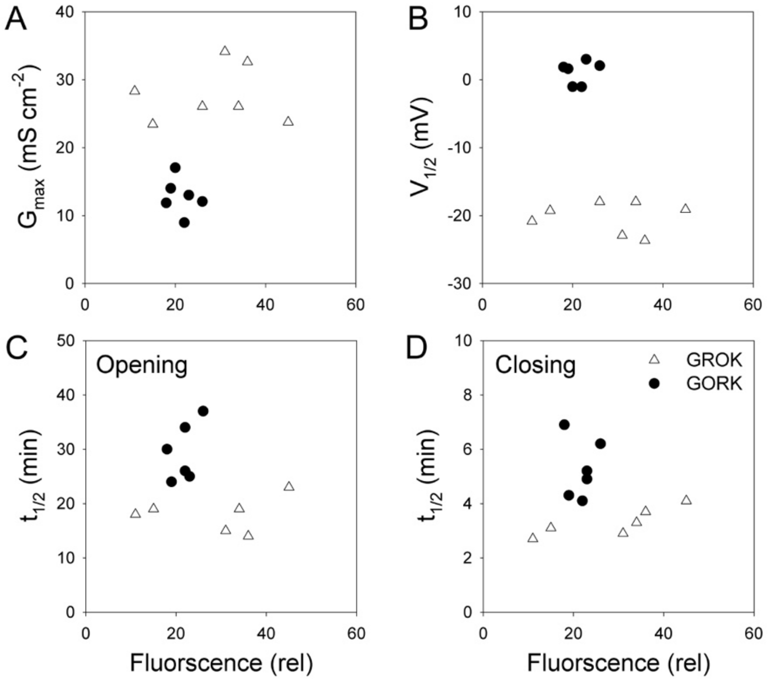

Supplementary Figure S8. Expression levels for the GROK- and GORK-complemented Arabidopsis do not correlate with phyisology

(A) Maximum conductance (G_{max}) and **(B)** conductance midpoint voltages ($V_{1/2}$) measured from guard cells superfused with 5 mM Ca2+-MES, pH 6.1, and 10 mM KCl, plotted against the GFP-fluorescence intensity for the data of Figure 4. Halftimes ($t_{1/2}$) for **(C)** opening and **(D)** closing of stomata plotted against the GFP-fluorescence intensity for the data of Figures 5 and 6.

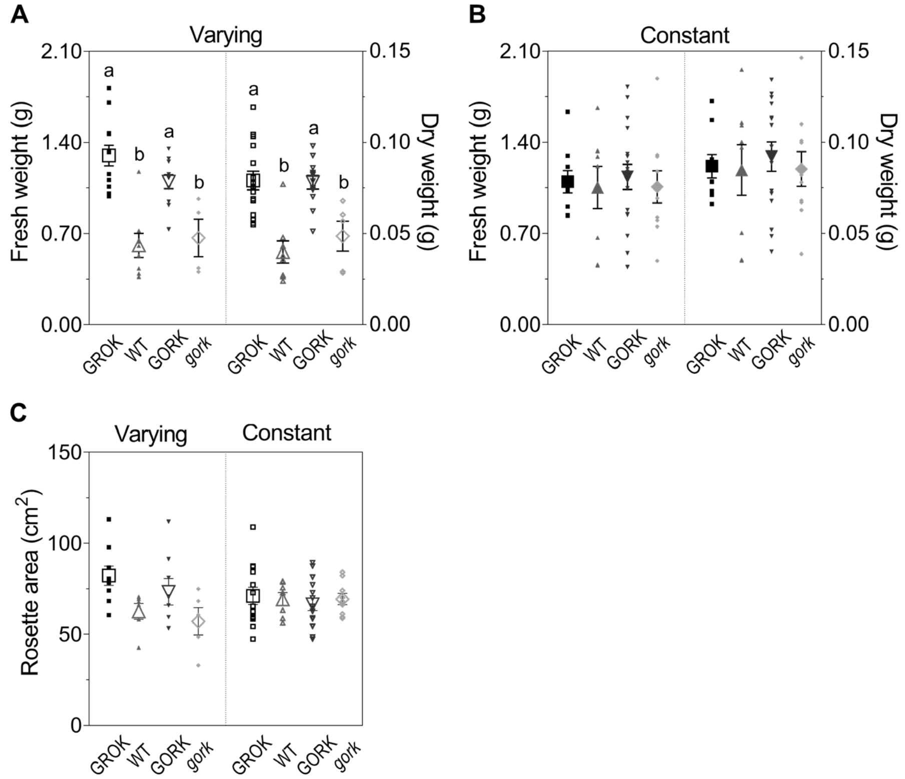

Supplementary Figure S9. GROK-complemented Arabidopsis show enhanced biomass yield under varying light when grown water-replete.

Fresh weight and dry weight under **(A)** varying (open symbols) or **(B)** constant light regime (filled symbols) of GROK complemented *gork* mutant (n=5), *Arabidopsis thaliana* wild-type (n=8), GORK complemented *gork* (n = 11) and *Arabidopsis thaliana* null *gork* mutant (n=8) plants. **(C)** rosette area under varying (open symbols) or constant light regime (filled symbols). Plants were harvested as in Figure 4. Plants maintained with 70±5% soil moisture throughout. Data are of individual transgenic lines (small symbols) and their means ±SE (large symbols) with letter indicating significant differences following post-hoc *Tukey* test ($p<0.05$).

Supplementary Table S1. Amino acid sequence identity matrix for *Gynandropsis gynandra* channels with voltage-gated K^+ channels of *Arabidopsis thaliana* and outward-rectifying K^+ channels of *Tarenyana hassleriana*.

Percentage of identity between sequences of putative K^+ channels from *Gynandropsis* and annotated K^+ channels of *T. hassleriana* and Arabidopsis. Matrix constructed with BioEdit 7.2. Light, medium, and dark gray highlights identities higher than 60%, 70% and 80%, respectively. Abbreviations: At = *Arabidopsis thaliana*, Gg = *Gynandropsis gynandra*, Th = *Tarenaya hassleriana*. Accession numbers: AtGORK = AT5G37500, AtSKOR = AT3G02850, AtKAT1 = AT5G46240, AtKAT2 = AT4G18290, AtAKT1 = AT2G26650, AtAKT5 = AT4G32500, AtAKT6 = AT2G25600, AtAKT2 = AT4G22200, AtKC1 = AT4G32650, ThGORK = XP_010549861.1, ThSKOR = XP_010525078.1. Full sequences of the Gynandropsis genes are listed in Appendix 2.

	Gyandropsis gynandra											
	13038	10030	22935	00763	25206	20501	16381	17148	04276	00873	26404	08174
AtGORK	77	68	67	24	21	26	23	21	19	24	15	23
AtSKOR	66	80	83	25	23	26	23	21	18	25	15	22
ThGORK	85	68	68	23	21	26	24	20	19	23	15	24
ThSKOR	68	85	88	24	21	26	23	20	18	25	15	22
AtKAT1	22	23	23	65	45	34	35	38	29	65	30	34
AtKAT2	21	22	23	69	47	35	35	38	29	67	31	35
AtAKT1	26	26	27	36	31	37	76	30	65	36	41	55
AtAKT5	25	25	26	35	30	35	56	28	47	34	32	74
AtAKT6	25	26	27	35	30	35	55	28	47	35	32	74
AtAKT2	25	26	26	36	34	72	35	30	29	35	24	36
AtKC1	21	20	20	34	31	29	28	69	22	32	25	28

Supplementary Table S2. List of constructs.

Vector	Gene	Use	Reference
pDONR207	Gg13038-wo	Entry clone	This paper
pUBC-Dest- GFP	pUBC-Gg13038-GFP	Localisation	This paper
pGT-Dest-GFP	pGT-Gg13038-GFP	Oocytes	This paper
pGC1C-DEST- GFP	pGC1C-Gg13038-GFP	Floral dip	This paper
p35S-Dest-GFP-T35S	p35S-Gg13038-GFP	Localisation	This paper
pGT-Dest-GFP	pGT-AtGORK-wo-GFP	Oocytes	(Horaruang et al., 2022)
pGC1C-DEST- GFP	pGC1C-AtGORK-GFP	Floral dip	(Horaruang et al., 2022)

Supplementary Appendix 1. Amino acids sequences of outwardly - rectifying K^+ channels of *Gynandropsis gynandra* genes

G. gynandra 13038

MMCLLRRCGRKDAEEQNGDVSTVGRRLSLVDKIRWIDLSDRRKFDREFLSGRFIIDPTTRW
YKTWTMFILVWALYSSLFTPMEFGFFRGLPENLFILDIVGQIAFLFDIVFQFFVAFRDSQTYRI
VSKPAPIAIRYLKSDFFPDLLSCLPWDLIFKASGRHEIVRYMLWIRLYRVRKVIVFFQRMEKD
TRINYLFSRIMKLLFVEVYCTHTAACVFYYLATTYPPEKEGYTWIGSLKLGDYSYENFRHIDI
WKRYITSLYFAIVTMATVGYGDIHAVNVREMIFVMIYVSFDMVLGAYLIGNITALIVKGSKTEK
FRDKMNDLLKYMNRNKLGRDIRSQITSHVRLQYESNYTDSALLQDMPVSIRAKIAQRLYMR
YIEKITLFKGCSTEFINQIVIRLHEEFFLPGEVIMEQGNVVDQLYFVCEGLLEVVGTERDGSED
PVTHLGPHDSFGDISIICNIPQPFTVRVCELCRLLRIDKQSFTDILEIYFHDGRKILDNLMEGKE
SNVRIKKLQSDIMMHIGKQEAELALKVNNAAYQGDLYQLKSLIRAGADPNKTDYDGRSPLHL
SAARGYEDITQFLVQEGVNINLKDKFGNTPLLEAVRNGQDDVIALLVREGATFDLEDTGNFL
CTAVAKGDSDFLRRLLSSGVDPNAKDYDHRTPLHVAASEGLYLMAKMLVEAGASVIAKDR
WGNSPLDEARMCGNKKLIKLLEDAKTVQSSIYPSSSHELQAERIHRRKCTVFPFHPHEIEEH
SRRHGVVVWIPSDLQQLISTAAQHLGLSDDSFIALSEDAGRITDTDMISDGQKLYLVATNQT
Y

Coding sequence

ATGATGTGTCTGCTGCGGAGGTGCGGAAGAAAGGATGCGGAGGAGCAAAACGGCGAC
GTATCTACGGTAGGGCGCAGATTGAGCTTGGTAGATAAGATCCGGTGGATCGATTTGT
CCGATCGTCGGAAATTCGACCGCGAATTCCTCTCCGGCAGATTCATCATCGATCCCACT
ACCAGGTGGTACAAGACATGGACGATGTTCATATTGGTGTGGGCACTCTACTCCTCTCT
TTTCACTCCCATGGAGTTTGGTTTCTTCCGCGGTCTCCCTGAGAATCTCTTCATCCTTGA
CATCGTTGGCCAAATCGCTTTCTTGTTCGATATCGTCTTTCAGTTCTTCGTCGCATTTAG
AGATAGTCAGACATATCGCATTGTCTCCAAACCTGCTCCCATCGCTATCCGGTACTTGA
AGTCGGACTTCTTCCCGGACTTGCTCAGTTGCTTACCTTGGGATCTCATTTTCAAGGCA
TCAGGGAGACACGAAATTGTGAGGTACATGTTGTGGATAAGGTTGTATCGGGTGCGGA
AGGTCATCGTATTCTTTCAAAGGATGGAGAAAGATACACGGATCAACTACCTCTTCAGT
AGAATCATGAAGCTATTGTTCGTTGAAGTGTATTGTACGCACACCGCAGCCTGCGTTTT
CTACTACTTGGCAACCACATATCCTCCGGAGAAAGAGGGCTACACTTGGATCGGAAGTT
TAAAGCTGGGGGATTACAGCTACGAGAATTTCAGACATATCGATATCTGGAAACGCTAT
ATTACATCTTTGTACTTTGCCATTGTCACTATGGCAACTGTGGGATATGGAGACATACAC
GCGGTGAATGTGAGAGAGATGATATTCGTAATGATATACGTTTCTTTTGATATGGTTCTC
GGGGCGTACCTGATTGGTAACATCACTGCTCTGATCGTCAAAGGATCAAAAACAGAGAA

ATTCAGAGACAAAATGAATGATCTGTTGAAGTACATGAACCGCAATAAGCTCGGGAGGG
ACATTCGCAGTCAGATAACAAGTCACGTTAGATTACAGTATGAGAGCAACTACACGGAC
AGCGCTCTTCTCCAGGACATGCCCGTATCTATTCGTGCAAAGATTGCGCAGAGGTTATA
CATGCGGTACATTGAGAAGATCACTCTATTCAAAGGATGCTCTACGGAGTTCATTAATC
AGATAGTCATTAGGCTCCATGAAGAGTTTTTTCTACCTGGAGAAGTAATAATGGAACAA
GGGAATGTTGTGGATCAGTTATATTTCGTCTGCGAAGGCTTACTGGAAGTTGTTGGGAC
GGAGAGAGATGGCTCAGAAGATCCTGTAACTCATTTAGGGCCTCACGACTCTTTCGGA
GATATCTCCATCATTTGCAACATCCCTCAGCCTTTCACTGTCCGCGTTTGTGAGCTCTGT
CGCCTCTTACGCATCGATAAACAGTCCTTCACGGATATCCTCGAGATTTATTTCCACGA
CGGACGCAAGATCTTGGATAATCTTATGGAGGGGAAGGAGTCAAATGTGAGGATCAAG
AAACTACAGTCAGATATTATGATGCACATTGGGAAACAAGAAGCGGAACTCGCTTTGAA
GGTAAACAATGCAGCTTATCAGGGAGACCTCTACCAGCTTAAGAGTTTGATCCGTGCTG
GGGCTGACCCCAACAAGACAGATTACGATGGAAGATCGCCTCTGCATCTGTCAGCCGC
TAGAGGTTACGAGGACATTACGCAGTTTCTCGTTCAAGAAGGTGTAAATATCAATCTCA
AAGATAAGTTCGGTAACACACCATTGCTAGAGGCAGTGAGGAACGGGCAAGATGATGT
GATTGCTCTGCTTGTTAGAGAAGGAGCCACGTTTGATCTAGAAGATACCGGAAACTTCT
TATGCACCGCTGTAGCAAAGGGTGATTCCGATTTTCTTAGGAGGCTTCTCTCCAGCGGC
GTTGACCCTAACGCCAAAGACTATGATCACAGAACGCCACTCCATGTCGCCGCATCTG
AAGGCTTGTACTTGATGGCTAAGATGCTTGTTGAAGCTGGAGCGAGCGTTATTGCCAAA
GACCGATGGGGAAATTCTCCGCTTGATGAAGCGCGGATGTGTGGTAACAAGAAACTGA
TTAAGTTACTCGAAGACGCGAAAACAGTTCAGTCTTCTATCTATCCTTCAAGCTCTCATG
AACTACAAGCGGAGAGGATTCACAGACGGAAATGTACGGTTTTCCCATTCCACCCACAC
GAGATTGAAGAGCATAGCAGAAGGCATGGAGTCGTGGTTTGGATCCCCAGCGATCTGC
AACAACTCATCTCAACCGCTGCACAACACCTTGGACTCTCCGACGATTCCTTCATTGCA
TTATCAGAAGACGCTGGTCGGATTACAGACACTGATATGATCAGCGATGGACAAAAGCT
GTATTTGGTCGCAACAAATCAAACATAC

G. gynandra 22935
MRNGDPPRSDDSEGDEDYEVDDLRDGIVSSRGSRFNLLSSFLGLDFAGNGRKTVFNGIRDI
SRGSIVHPDNRWYKAWTMFILVWALYSSFFTPLEFGFFRGLPENLFILDIVGQVAFLVDIVLQ
FFVAYRDSRTYRMVYRRSSIAIRYLKSSFVIDLLACMPWDNIYRAVGEKEEVRYLLWIRLYRV
NKLVDFFQKMEKDIRINYLFTRIVKLIFVELYCTHTAACIFYYLATTLPASQEGSTWIGSLKMG
DYSYSSFRDIDLWTRYTTSMYFAIATMATVGYGDIHAVNVREMIFVMVYISFDMILGAYLIGN
MTALIVKGSKTEKFRDKMADIMKYMNRNKLGRGIRSQITGHFRLQYESSYTEAAALQDIPISI
RAKIAQTLYMPYLEKIPLFNGCSSEFINQIVIRLHEEFFLPGEVILEQGSVVDQLYFVCHGVLE
EIGTAKDGSEEIVALLQPDSSFGEISILCNIPQPYTVRVSELCRLLRLDKQSFMNILEIFFHDG
RKILNNLLEGKESNVRIKQLESDITYHISKQEAELALKVNSAAYYGDLYQLKSLIRAGADPNKT

DYDGRSPLHLAASRGYEDIMLYLIQEGVDVNFKDKLGNTPLLEAIKNGNDRVASLLVKEGAS
LEIENGGMFLCSVVVKGDSDLLKRLLENGIDPNSKDYDHRTPLHVAASEGLYLMAKQLVQA
GAFVLAKDRWGNTPLDEALACGNKSLIKLLEAAKHSQISSFPSSSKELKEKMKRKKCTVYPL
HKKRGVVLWVPESMEELIKTAAEQLQIPGASYVLSEDEAIILDIDMINDGQKLYLTFHNSLY

Coding sequence

**ATGAGAAACGGAGATCCTCCCCGGAGCGACGATTCCGAGGGGGATGAGGATTACGA
GGTGGATGATCTGAGGGATGGAATAGTTTCGTCGCGTGGCAGCAGATTTAACCTCCT
CTCCAGTTTTTTAGGGTTAGACTTCGCCGGAAACGGCCGGAAAACCGTCTTCAATGGT
ATCAGAGACATTTCCAGAGGCTCCATCGTCCATCCCGATAACCGGTGGTACAAGGCA
TGGACGATGTTCATATTGGTGTGGGCACTTTACTCTTCTTTCTTCACTCCCTTGGAGTT
TGGCTTCTTCAGAGGATTGCCAGAGAATCTCTTTATTCTGGACATTGTTGGACAAGTG
GCTTTTTTAGTAGACATTGTTTTGCAATTCTTCGTTGCGTATAGAGATAGTCGCACGTA
TAGAATGGTGTATAGGCGCAGCTCTATCGCTATAAGGTACTTGAAATCAAGTTTTGTC
ATTGATTTACTCGCTTGCATGCCATGGGATAACATCTACAGAGCTGTAGGTGAAAAAG
AAGAAGTGAGATACCTATTGTGGATCAGGTTATATCGGGTGAACAAACTCGTAGACTT
TTTCCAGAAAATGGAAAAGGATATACGAATTAATTACCTATTCACTAGGATTGTCAAA
CTTATATTCGTTGAGCTCTATTGCACTCACACCGCAGCCTGTATATTCTATTACTTAGC
CACCACTCTGCCTGCTTCCCAAGAAGGATCCACATGGATTGGAAGCTTGAAGATGGG
TGATTACAGTTATTCTAGTTTCAGAGATATCGATCTTTGGACACGATATACAACTTCCA
TGTATTTTGCAATTGCTACAATGGCAACTGTTGGTTATGGAGATATACATGCTGTGAAT
GTACGGGAAATGATATTTGTAATGGTCTATATCTCATTCGACATGATTCTAGGTGCTTA
TTTGATTGGTAACATGACGGCTTTAATAGTTAAAGGCTCAAAAACAGAAAAATTCCGG
GACAAAATGGCGGATATTATGAAGTATATGAACAGAAACAAACTTGGGAGAGGCATC
CGCAGTCAAATCACTGGCCATTTCCGTTTACAGTATGAGAGTAGCTACACTGAAGCA
GCTGCGCTTCAGGATATACCGATATCTATTCGTGCTAAGATTGCACAAACTTTATACA
TGCCTTATCTTGAAAAGATTCCCCTCTTCAACGGGTGTTCATCTGAATTCATAAATCAA
ATCGTCATAAGATTACATGAAGAGTTTTTCCTCCCAGGAGAGGTTATATTAGAGCAAG
GGAGCGTTGTGGATCAATTATACTTCGTTTGTCATGGTGTACTGGAGGAGATAGGTAC
AGCCAAGGACGGATCAGAAGAGATAGTGGCACTTTTACAGCCGGATAGTTCTTTCGG
GGAAATTTCAATCCTCTGTAATATTCCTCAACCTTACACAGTTCGGGTTTCTGAACTAT
GCCGGCTTTTAAGACTTGATAAGCAGTCTTTTATGAATATTCTCGAGATATTTTTTCAC
GATGGGCGAAAAATCCTAAACAATCTTCTCGAGGGAAAAGAATCAAATGTTCGGATT
AAGCAACTGGAGTCCGATATTACATATCACATAAGTAAGCAAGAGGCAGAACTGGCG
TTGAAGGTGAATAGTGCGGCTTACTATGGAGACCTTTACCAGCTTAAAAGTCTGATCC
GAGCAGGAGCTGATCCGAATAAGACTGATTATGATGGAAGATCGCCTTTGCATCTTG
CTGCCTCTAGAGGATATGAAGACATCATGCTCTATCTCATTCAAGAAGGAGTAGATGT**

TAACTTCAAAGATAAATTAGGAAACACGCCATTGTTAGAAGCTATAAAGAACGGGAA
TGATCGCGTGGCGAGTTTACTAGTGAAAGAGGGTGCCTCACTCGAAATCGAGAATGG
AGGGATGTTCCTTTGCTCAGTGGTGGTAAAGGGCGACAGTGATTTGCTCAAACGACTT
TTGGAGAATGGAATTGATCCGAATTCTAAAGATTATGATCACAGGACACCTCTTCATG
TCGCTGCATCGGAAGGACTATACCTTATGGCAAAGCAGTTGGTCCAGGCTGGTGCTT
TTGTTCTCGCAAAGGACAGATGGGGGAATACTCCTCTGGATGAAGCCTTGGCTTGTG
GGAACAAGAGCTTGATCAAACTACTCGAAGCTGCCAAACATTCTCAAATATCTTCATT
TCCAAGTAGCTCTAAAGAACTCAAAGAGAAGATGAAGAGGAAGAAATGTACAGTGTA
TCCATTGCATAAAAAACGAGGGGTGGTATTATGGGTGCCGGAGAGTATGGAGGAGCT
GATAAAGACAGCGGCGGAGCAACTGCAGATCCCCGGAGCTTCCTACGTCTTGTCGGA
AGATGAAGCTATCATTCTCGACATTGATATGATTAATGATGGACAGAAACTTTATTTAA
CATTCCATAATTCTCTCTAT

G. gynandra 10030
MGNERHRRRGESEGEEEYEVEDLRDEIVSSRGSRFNLLSTFLGLDFVGNGRNNVFIGIRNI
SIGRIVHPDNRWYKTWTIFILVWALYSSFFTPLEFGFFRGLPNNLFILDIVGQVAFLLDIVLQFF
LAYRDSRTYRMVYRHSSIAIRYLKSSFFIDLLACMPWDTIYKCTGEKEEVRYLLWIRLYRVNK
LVHFFHKMEKDIRINYQFTRIVKLLFVELYCTHTAACIFYYLATTLPASQEGYTWIGSLKMGD
HSYSSFRDIDIWTRYTTSMYFAIATMATVGYGDIHAVNVREMIFVMVFISFDMILGAYLIGNM
TALIVKGSKTEMFRDKMADIMKYMNRNKLGRDIRDQISGHFRLQYESSYTEAAALQDIPMSI
RAKIAQTLYMLHIEKIPLFKGCSSEFINQIVIRLHEEFFLPGEVILEQGSVVDQLYFVCHGVLEE
IGTIKDGSEELVALLQPDSSFGEISILCNIPQPYTVRVSELCRLLRLDKQSFMNILEIYFHDGRK
ILNNLLEGKESNLRIKQLESDITYHIGKQEAELALKVNSAAYYGDLYQLKSLIRAGADPNKTDY
DGRSPLHLSASRGYEDVTLYLIQEGVDVNLKDKLGNTALLEAIKNGNDGVACLLVKEGASM
DIDNAGTFLCTVVAKGDSDFLKRLLENGTDPNSKDYDHRTPLHVAASEGLNLMAKQLVQAG
AFVLAKDRWGNTPLDEALSCGNKNLIKLLEDAKTSQISSFPTTSRELKDKIQRKKKCTVYPF
HPNEPKEKRKVGVVLWVPQSMAELVKAAGEQLEIPDPWCLLSEDEGKINDVDMINDGQKL
YLTVDQSPNP

Coding sequence
ATGGGGAACGAAAGGCATCGTCGGAGAGGAGAATCAGAAGGCGAGGAGGAGTACGAG
GTGGAGGATCTGAGGGATGAGATTGTTTCGTCTCGAGGCAGCAGATTCAACCTTCTCT
CCACTTTCTTAGGGTTAGACTTCGTCGGAAATGGTCGAAATAATGTCTTCATTGGAATCA
GAAACATCTCCATAGGCCGCATTGTCCACCCCGATAACCGATGGTACAAGACATGGAC
GATTTTCATATTGGTATGGGCACTTTACTCTTCTTTCTTCACTCCATTGGAGTTTGGCTTT
TTCAGAGGTTTACCAAACAACCTCTTTATTCTCGACATTGTTGGGCAAGTGGCATTTTTA
TTAGACATTGTTTTGCAATTCTTCCTGGCCTATAGAGACAGTCGCACTTACAGAATGGTG

TATAGACACAGCTCCATTGCTATACGGTACTTGAAATCGAGTTTTTTTATCGATTTACTC
GCTTGCATGCCGTGGGACACCATATACAAGTGTACAGGTGAAAAAGAAGAAGTAAGATA
CTTATTGTGGATTAGGTTATATAGGGTGAACAAACTCGTCCATTTTTTCCATAAAATGGA
GAAGGATATACGAATAAATTACCAGTTCACTCGGATTGTCAAGCTTTTGTTCGTCGAGCT
CTATTGCACTCATACTGCAGCCTGCATCTTCTACTACTTGGCAACGACTCTGCCTGCTT
CTCAAGAAGGGTACACGTGGATTGGAAGCCTGAAGATGGGCGATCATAGTTATTCTAGT
TTCAGAGATATCGATATTTGGACAAGATATACAACTTCCATGTATTTTGCAATCGCCACA
ATGGCTACTGTTGGTTATGGAGATATACATGCTGTGAACGTGCGGGAAATGATATTCGT
AATGGTCTTCATTTCATTCGACATGATTCTTGGAGCTTATTTGATCGGTAACATGACAGC
TCTGATTGTTAAAGGATCAAAAACAGAAATGTTTCGGGATAAAATGGCCGATATTATGAA
GTATATGAATAGAAACAAACTGGGGAGAGACATCCGTGATCAAATCAGTGGCCATTTCC
GCTTGCAGTACGAAAGCAGCTACACCGAGGCAGCTGCTCTTCAAGATATACCGATGTC
TATTCGTGCTAAGATTGCGCAGACTTTATACATGCTGCATATCGAAAAGATCCCCCTCTT
CAAGGGATGCTCATCAGAGTTCATAAATCAAATTGTCATTAGACTTCATGAAGAGTTTTT
CCTCCCAGGAGAAGTTATCTTGGAGCAAGGAAGTGTCGTGGATCAATTGTACTTCGTTT
GTCACGGTGTACTCGAGGAGATTGGAACGATAAAGGATGGATCGGAGGAATTAGTGGC
GCTTTTACAGCCAGATAGTTCGTTTGGTGAAATTTCGATCCTGTGTAATATTCCTCAACC
TTACACGGTTCGGGTTTCTGAACTCTGTCGTCTTCTAAGGCTGGATAAGCAATCTTTTAT
GAATATTCTTGAGATATATTTTCACGATGGACGAAAAATCCTTAACAATCTCCTCGAGGG
AAAAGAATCGAACCTACGGATTAAGCAACTTGAATCCGATATTACTTATCATATCGGTAA
GCAAGAGGCAGAACTCGCGTTGAAGGTGAATAGCGCTGCCTACTATGGAGATCTCTAC
CAACTTAAAAGTCTGATCCGAGCTGGGGCTGACCCAAATAAGACTGATTATGACGGGA
GGTCGCCTTTGCATCTCTCTGCCTCTAGAGGATACGAGGATGTTACGCTCTATCTTATT
CAAGAAGGAGTCGACGTAAACTTGAAAGATAAACTAGGAAACACAGCGTTGCTTGAAGC
CATTAAGAACGGGAATGATGGAGTGGCATGTTTACTAGTGAAAGAAGGGGCCTCAATG
GATATAGACAACGCAGGAACATTCTTATGCACAGTCGTTGCAAAGGGTGATAGTGACTT
TCTCAAACGGCTTTTGGAAAATGGAACTGATCCAAATTCGAAAGACTATGATCACAGAA
CGCCTCTTCATGTAGCTGCATCCGAAGGGTTGAACCTTATGGCAAAGCAGTTGGTCCA
GGCTGGTGCTTTTGTTCTTGCAAAGGACAGATGGGGGAATACTCCTCTGGATGAAGCC
TTGTCCTGCGGGAATAAGAACTTGATAAAACTACTCGAAGACGCTAAAACTTCTCAGATT
TCATCATTTCCGACCACTTCCAGAGAACTAAAAGATAAGATTCAGCGGAAGAAGAAATG
TACGGTGTACCCTTTCCATCCAAACGAGCCTAAAGAGAAGAGGAAAGTCGGGGTCGTA
TTGTGGGTCCCGCAAAGCATGGCAGAGCTGGTTAAAGCCGCCGGCGAACAACTTGAGA
TTCCAGATCCTTGGTGCTTATTGTCGGAAGATGAAGGTAAGATTAACGATGTTGATATG
ATTAACGATGGTCAGAAACTGTATTTAACCGTTGATCAATCTCCAAATCCA

Supplementary Appendix 2. OnGuard3 parameter set highlighting GORK and mGROK gating

Parameter adjustments for the *gork* mutant are indicated in ***bold italics***. Adjustments for the shift in mGROK $V_{1/2}$ are shown in **bold**.

Total cell volume = 0.381545 pL; vacuolar fraction = 79.2181%

Stomatal Aperture Parameters:
SA:P 'm'= 0.8 atm/µm; SA:P 'n'= 3 atm; SA:V 'R'= 0.05 pL/µm SA:V 'S'= 0.3 pL
Pore Length: 8 µm; Pore Depth: 15 µm

Cytosolic Protein Buffering: [Pr] = 0.143864 mM, pIso = 6.8, ap = -71
Cytosolic Calcium Buffering: [Bu] = 0.318061 mM, K = 3e-06 M # Ca Sites = 10

Temperature: Leaf: 25∞C; Air: 25∞C
Current 'time' in model = 00:00:00.00

*** Compartmental Solutions (/mM)
==================================

	Apoplast	Cytosol	Vacuole
pH	6.500000	7.689380	5.286889
K	10.00000	107.7377	26.52061
Ca	1.000000	0.012759	40.24475
Cl	12.00000	15.93618	64.75251
Suc	0.010000	0.029115	0.004663
MH2	3.23e-07	2.89e-07	0.314942
MH	7.94e-06	0.000110	0.473281
M	0.009992	2.138469	36.47602
HCO3	0.022243	0.344017	0.001362
CO2	0.015807	0.015807	0.015807
Malates	0.010000	2.138579	37.26425

*** Plasma Membrane Transporters
=================================

PM K-in Channel [3000 units] (Inward-Rectifying GHK Channel)

#'K' G/Gmax = 9 pOhms
      2-State Voltage Gate: VΩ = -185 mV,
      Zg = +1.8
Light-Sensitive: NO!
Ligand-Gates:
  Ca-inhibited (cytosol): Kd= 3.3e-07, Hill= 4;
  H-activated (cytosol): Kd= 6e-08, Hill= 2;
  H-activated (apoplast): Kd= 1e-07, Hill= 1;

PM K-out Channel [240 units or ***0 units***] (Outward-Rectifying GHK Channel)

#'K' G/Gmax = 20 pOhms
      VΩ = +1 ◊ F/RT ◊ ln([K]apo/10mM) or +1 ◊ F/RT ◊ ln([K]apo/**40mM**)
      Zg = +2
Light-Sensitive: NO!
Ligand-Gates:
  H-inhibited (cytosol): Kd= 3e-08, Hill= 2;
  HCO3-activated (cytosol): Kd= 0.0003, Hill= 2;

R-Type Anion Channel [1000 units] (Outward-Rectifying GHK Channel)

#'Cl' G/Gmax = 3.4 pOhms
#'M' G/Gmax = 2 pOhms
      VΩ = +1 ◊ F/RT ◊ ln(1e-06mM/[H]cyt)
      Zg = -2
Light-Sensitive: NO!
Ligand-Gates:
  Ca-activated (cytosol): Kd= 6e-07, Hill= 4;
  H-activated (cytosol): Kd= 3e-08, Hill= 3;
  HCO3-activated (cytosol): Kd= 0.0003, Hill= 3;

```
V-Gated Ca-IN [12 units] (Inward-Rectifying GHK Channel)
-------------
#'Ca' G/Gmax = 12 pOhms
      VΩ = +0.5 ◊ F/RT ◊ ln(2e-09mM/[Ca]cyt)
      Zg = +1
Light-Sensitive: NO!
Ligand-Gates:
  Ca-inhibited (cytosol): Kd= 5e-07, Hill= 5;


Anion VIC [300 units] (Outward-Rectifying Ohmic Channel)
---------
#'Cl' G/Gmax = 0.15 pOhms
#'M'  G/Gmax = 0.07 pOhms
      Voltage-Independent
Light-Sensitive: NO!
Ligand-Gates:
  Ca-activated (cytosol): Kd= 6e-07, Hill= 4;
  H-activated (cytosol): Kd= 4e-08, Hill= 2;
  HCO3-activated (cytosol): Kd= 0.0003, Hill= 1;


H-ATPase [300000 units] (4-State 'Slayman' Pump)
--------
#'H'  Stoichiometry = +1; binds at 4->1 (in) and 3->2 (ex);
      K12 = 2000, K23 = 50000,     K34 = 500,  K41 = 2e+09,
      K21 = 100,  K32 = 1e+08,     K43 = 10,   K14 = 200;
Light-Sensitive: Yes: LΩ = 50 µEinsteins, Fmin = 5%
Ligand-Gates:
  Ca-inhibited (cytosol): Kd= 2.5e-07, Hill= 3;


H:Cl Symport [50000 units] (4-State 'Slayman' Pump)
------------
#'H'  Stoichiometry = +2; binds at 4->1 (in) and 3->2 (ex);
#'Cl' Stoichiometry = +1; binds at 4->1 (in) and 4->3 (ex);
      K12 = 1000, K23 = 100,  K34 = 50000,     K41 = 1e+21,
      K21 = 50,   K32 = 1e+21,     K43 = 100000,    K14 = 100;
```

```
Light-Sensitive: NO!
Ligand-Gates:
 <none>


H:K Symport [16000 units] (4-State 'Slayman' Pump)
-----------
#'H'  Stoichiometry = +1; binds at 4->1 (in) and 3->2 (ex);
#'K'  Stoichiometry = +1; binds at 4->1 (in) and 4->3 (ex);
      K12 = 2,    K23 = 10000,     K34 = 100000,    K41 = 1e+14,
      K21 = 0.4,  K32 = 1e+12,     K43 = 1e+10,     K14 = 50;
Light-Sensitive: NO!
Ligand-Gates:
 <none>


Ca-ATPase [60000 units] (4-State 'Slayman' Pump)
---------
#'Ca' Stoichiometry = +1; binds at 4->1 (in) and 3->2 (ex);
      K12 = 2000, K23 = 10000,     K34 = 500,  K41 = 1e+15,
      K21 = 2,    K32 = 1e+07,     K43 = 500,  K14 = 1000;
Light-Sensitive: Yes: LΩ = 50 µEinsteins, Fmin = 50%
Ligand-Gates:
  Ca-activated (cytosol): Kd= 5e-07, Hill= 2;
  HCO3-inhibited (cytosol): Kd= 0.0003, Hill= 4;


HMal symp [40000 units] (Concentration-Driven SYMPORT)
---------
#'H'  (Stoichiometry = -3)
#'M'  (Stoichiometry = -1)
      Fmax = 1e+20
Light-Sensitive: NO!
Ligand-Gates:
 <none>


K leak [10 units] (Outward-Rectifying GHK Channel)
------
```

```
#'K'  G/Gmax = 10 pOhms
      VΩ = +1 ◊ F/RT ◊ ln([K]apo/1mM)
      Zg = +1
Light-Sensitive: NO!
Ligand-Gates:
 <none>


*** Tonoplast Transporters
==========================

TPK1 [300 units] (Inward-Rectifying Ohmic Channel)
----
#'K'  G/Gmax = 90 pOhms
      Voltage-Independent
Light-Sensitive: NO!
Ligand-Gates:
  Ca-activated (cytosol): Kd= 3e-06, Hill= 1;
  H-activated (cytosol): Kd= 3e-08, Hill= 3;


TPC1 [100 units] (Outward-Rectifying GHK Channel)
----
#'Ca' G/Gmax = 27 pOhms
#'K'  G/Gmax = 14 pOhms
      VΩ = +1 ◊ F/RT ◊ ln([Ca]vac/2mM)
      Zg = +2
Light-Sensitive: NO!
Ligand-Gates:
  Ca-activated (cytosol): Kd= 3e-05, Hill= 1;
  H-activated (vacuole): Kd= 1e-06, Hill= 1;


FV K Channel [1600 units] (Inward-Rectifying GHK Channel)
------------
#'K'  G/Gmax = 6 pOhms
      2-State Voltage Gate: VΩ = -30 mV,
      Zg = +1
Light-Sensitive: NO!
```

```
Ligand-Gates:
  Ca-inhibited (cytosol): Kd= 2e-07, Hill= 1;
  H-inhibited (cytosol): Kd= 4e-07, Hill= 1;


VCL [300 units] (Inward-Rectifying GHK Channel)
---
#'Cl' G/Gmax = 40 pOhms
#'M'  G/Gmax = 10 pOhms
      VΩ = +1 ◊ F/RT ◊ ln([H]vac/0.005mM)
      Zg = -1
Light-Sensitive: NO!
Ligand-Gates:
  Ca-activated (cytosol): Kd= 1e-06, Hill= 1;


Vacuole H-ATPase [400000 units] (4-State 'Slayman' Pump)
----------------
#'H'  Stoichiometry = +2; binds at 4->1 (in) and 3->2 (ex);
      K12 = 100,  K23 = 1000, K34 = 0.5,  K41 = 1e+18,
      K21 = 10,   K32 = 1e+08,     K43 = 5,    K14 = 10000;
Light-Sensitive: Yes: LΩ = 50 µEinsteins, Fmin = 10%
Ligand-Gates:
 <none>


Vacuole H-PPase [1200000 units] (4-State 'Slayman' Pump)
---------------
#'H'  Stoichiometry = +1; binds at 4->1 (in) and 3->2 (ex);
      K12 = 1000, K23 = 1000, K34 = 1e+11,     K41 = 3e+09,
      K21 = 100,  K32 = 5e+09,     K43 = 1e+07,     K14 = 10000;
Light-Sensitive: Yes: LΩ = 50 µEinsteins, Fmin = 10%
Ligand-Gates:
  Ca-inhibited (cytosol): Kd= 1e-07, Hill= 1;
  K-activated (cytosol): Kd= 0.05, Hill= 1;


Slaymanesque Ca Pump [800000 units] (4-State 'Slayman' Pump)
--------------------
```

```
#'Ca' Stoichiometry = +1; binds at 4->1 (in) and 3->2 (ex);
      K12 = 3000, K23 = 1000, K34 = 1000, K41 = 1e+09,
      K21 = 0.3,  K32 = 10000,      K43 = 10,   K14 = 10000;
Light-Sensitive: Yes: LΩ = 50 µEinsteins, Fmin = 50%
Ligand-Gates:
  Ca-activated (cytosol): Kd= 3.5e-07, Hill= 3;
  HCO3-inhibited (cytosol): Kd= 0.0003, Hill= 4;
  Ca-inhibited (vacuole): Kd= 0.04, Hill= 4;


Vac.CLC [120000 units] (4-State 'Slayman' Pump)
-------
#'H'  Stoichiometry = +1; binds at 4->1 (in) and 3->2 (ex);
#'Cl' Stoichiometry = -2; binds at 1->4 (in) and 2->3 (ex);
      K12 = 1000, K23 = 1e+09,      K34 = 100,  K41 = 1e+10,
      K21 = 1000, K32 = 1e+09,      K43 = 10,   K14 = 1e+11;
Light-Sensitive: NO!
Ligand-Gates:
  H-inhibited (cytosol): Kd= 5e-08, Hill= 2;


Tonoplast VCa [8 units] (Outward-Rectifying GHK Channel)
-------------
#'Ca' G/Gmax = 10 pOhms
      VΩ = +1 ◊ F/RT ◊ ln(10mM/[Ca]vac)
         + +0.5 ◊ F/RT ◊ ln([HCO3]cyt/0.15mM)
      Zg = +4
Light-Sensitive: NO!
Ligand-Gates:
  Ca-activated (cytosol): Kd= 5e-07, Hill= 4;
T-deactivation: switch= [Ca]cyt; Threshold= 0.001mM, TΩ= 100000ms,
reset= 5%/0.0005mM


CAX [100000 units] (Concentration-Driven ANTIPORT)
---
#'H'  (Stoichiometry = -3)
#'Ca' (Stoichiometry = +1)
      Fmax = 1e+22
```

```
Light-Sensitive: NO!
Ligand-Gates:
  Ca-activated (cytosol): Kd= 3e-06, Hill= 1;


ALMT-Mal [600 units] (Inward-Rectifying GHK Channel)
--------
#'M'  G/Gmax = 6 pOhms
      2-State Voltage Gate: VΩ = +0 mV,
      Zg = -2
Light-Sensitive: NO!
Ligand-Gates:
  H-inhibited (cytosol): Kd= 8.5e-08, Hill= 2;
  Ca-activated (cytosol): Kd= 1e-06, Hill= 1;


NHX [20000 units] (Concentration-Driven ANTIPORT)
---
#'H'  (Stoichiometry = -1)
#'K'  (Stoichiometry = +1)
      Fmax = 100000
Light-Sensitive: NO!
Ligand-Gates:
 <none>


Vac MLC [0 units] (4-State 'Slayman' Pump)
-------
#'H'  Stoichiometry = +1; binds at 4->1 (in) and 3->2 (ex);
#'M'  Stoichiometry = -1; binds at 1->4 (in) and 2->3 (ex);
      K12 = 50000,     K23 = 2e+07,     K34 = 100,  K41 = 1e+10,
      K21 = 1000, K32 = 1e+09,     K43 = 10,   K14 = 1e+11;
Light-Sensitive: NO!
Ligand-Gates:
  H-inhibited (cytosol): Kd= 5e-08, Hill= 2;


*** METABOLISM
==============
```

Total Malate (apo/cyt/vac) = 0.01 2.13858 37.2642 mM
Total Sucrose (apo/cyt/vac) = 0.01 0.0291147 0.00466311 mM

Photosynthesis:
Suc s-max = 10 fmol/h, LΩ = 50 µE
Mal s-max = 0 fmol/h, LΩ = 50 µE
Light Type: Total

Sucrose Sink:
R-max = 10 fmol/h, KΩ = 1 mM

Suc <-> Mal Conversion:
R-max = 5 fmol/h, KΩ(S) = 0.1 mM, KΩ(M) = 10 mM
Mid-point pH = 7.7, pH gradient = +64

'Q10' Temperature Coefficient: 2

*** PHOTOSYNTHESIS & WUE PARAMETERS
===================================

Stomata/mm≤ = 100
Stomatal Length/µm = 8; Depth = 15
Subepidermal Depth/µm: = 300; Empty Space = 50%

CO2 Assimilation, A = AL ◊ AC - Rd [Where AL = {fL + Amax-v[(fL + Amax)≤-4T.fL.Amax]}/2T and AC = 1/[1 + Kc/(Cin-Cc)])]

Amax (µmol/m≤/s) = 20
f = 0.15; T = 0.9
Kc (/ppm) = 80; Cc (/ppm) = 8; Rd (µmol/m≤/s) = 3

System used for Ciso = Mott (p-site)
Wet Surface Area Coefficient (RWF) = 40

*** Constraint Relaxation & Recovery
====================================

Use CRR? Yes

Solutes to include: K

Nominal Capacity (Amax, /fmol) = 14

Second Capacity (Smax, /fmol) = 0.5

Max Recovery Rate (/fmol/sec) = 0.006; (Order = 1)

Current Contents = 100.0%

Apply Turgour-Sensitivity? Yes

Sigmoid Mid-Point (atm) = 7; Sigmoid Gradient (/atm) = 2
